# Effects of a Family-Based Lifestyle Intervention Plus Supervised Exercise Training on Abdominal Fat Depots in Children With Overweight or Obesity: A Secondary Analysis of a Nonrandomized Clinical Trial

**DOI:** 10.1001/jamanetworkopen.2022.43864

**Published:** 2022-11-28

**Authors:** Cristina Cadenas-Sanchez, Rafael Cabeza, Fernando Idoate, Maddi Osés, María Medrano, Arantxa Villanueva, Lide Arenaza, Aritz Sanz, Francisco B. Ortega, Jonatan R. Ruiz, Idoia Labayen

**Affiliations:** 1 Institute for Sustainability & Food Chain Innovation, Department of Health Sciences, Public University of Navarre, Pamplona, Spain; 2 Navarra Institute for Health Research, Pamplona, Spain; 3 PROFITH (Promoting Fitness and Health Through Physical Activity) Research Group, Sport and Health University Research Institute, Department of Physical and Sports Education, Faculty of Sport Sciences, University of Granada, Granada, Spain; 4 Centro de Investigación Biomédica en Red Fisiopatología de la Obesidad y Nutrición (CIBERobn), Instituto de Salud Carlos III, Madrid, Spain; 5 Department of Electrical, Electronic and Communications Engineering, Public University of Navarre, Pamplona, Spain.; 6 Department of Radiology, Mutua Navarra, Pamplona, Spain; 7 Department of Health Sciences, Public University of Navarre, Pamplona, Spain; 8 Smart Cities Institute, Public University of Navarre, Pamplona, Spain; 9 Faculty of Sport and Health Sciences, University of Jyväskylä, Jyväskylä, Finland; 10 Instituto de Investigación Biosanitaria, ibs.Granada, Granada, Spain

## Abstract

**Importance:**

Excess abdominal fat is a major determinant in the development of insulin resistance and other metabolic disorders. Increased visceral adipose tissue (VAT) seems to precede the development of insulin resistance and is therefore a prime target of childhood lifestyle interventions aimed at preventing diabetes.

**Objectives:**

To examine the effect of added exercise to a family-based lifestyle intervention program designed to reduce VAT plus subcutaneous (ASAT), intermuscular (IMAAT), and pancreatic (PAT) adipose tissue in children with overweight or obesity and to explore the effect of changes in VAT on insulin resistance.

**Design, Setting, and Participants:**

This 2-group, parallel-design clinical trial was conducted in Vitoria-Gasteiz, Spain. A total of 116 children with overweight or obesity participated and were assigned to a 22-week family-based lifestyle program (control group [n = 57]) or the same program plus an exercise intervention (exercise group [n = 59]). Data were collected between September 1, 2014, and June 30, 2017, and imaging processing for fat depot assessments and data analysis were performed between May 1, 2019, and February 12, 2021.

**Interventions:**

The compared interventions consisted of a family-based lifestyle and psychoeducation program (two 90-minute sessions per month) and the same program plus supervised exercise (three 90-minute sessions per week).

**Main Outcomes and Measures:**

The primary outcome of this study was the change in VAT between baseline and 22 weeks as estimated by magnetic resonance imaging. The secondary outcomes were changes in ASAT, IMAAT, and PAT. The effect of changes in VAT area on insulin resistance was also recorded.

**Results:**

The 116 participants included in the analysis (62 girls [53.4%]) had a mean (SD) age of 10.6 (1.1) years, and 67 (57.8%) presented with obesity. Significantly greater reductions were recorded for the exercise group in terms of reduction in VAT (−18.1% vs −8.5% for the control group; *P* = .004), ASAT (−9.9% vs −3.0%; *P* = .001), and IMAAT (−6.0% vs −2.6%; *P* = .02) fat fractions compared with the control group. Changes in VAT explained 87.6% of the improvement seen in insulin resistance (β = −0.102 [95% CI, −0.230 to −0.002]).

**Conclusions and Relevance:**

These findings suggest that the addition of exercise to a lifestyle intervention program substantially enhanced the positive effects on abdominal fat depots in children with overweight or obesity. In addition, the reduction in VAT seemed to largely mediate the improvement of insulin sensitivity. These results highlight the importance of including exercise as part of lifestyle therapies aimed at treating childhood obesity and preventing the development of type 2 diabetes.

**Trial Registration:**

ClinicalTrials.gov Identifier: NCT02258126

## Key Points

**Question:**

Does the addition of exercise to a family-based lifestyle program reduce abdominal fat depots in children with overweight or obesity?

**Findings:**

In this secondary analysis of a 22-week nonrandomized controlled trial of 116 participants, the addition of exercise to a lifestyle intervention resulted in clinically meaningful reductions in visceral adipose tissue and other abdominal fat depots in children with overweight or obesity. The reduction in visceral adipose tissue might mediate the improvement of insulin resistance.

**Meaning:**

These findings suggest that multicomponent therapies including a family-based lifestyle intervention and exercise can result in robust reductions in the size of abdominal fat depots, leading to insulin sensitivity benefits for children with overweight or obesity.

## Teaser

This secondary analysis of a nonrandomized clinical trial compares the association of a family-based lifestyle and psychoeducation program vs the same program plus supervised exercise training with abdominal fat depots in children aged 8 to 12 years with overweight or obesity.

## Introduction

Excess abdominal fat is a major determinant in the development of insulin resistance and other metabolic disorders.^1,2,3^ Visceral adipose tissue (VAT) is one of the most worrisome of all ectopic fat depots,^3^ having been associated with a 36% to 83% greater risk of all-cause mortality.^4,5,6^ Increased VAT seems to precede the development of insulin resistance^7^ and is therefore a prime target of childhood lifestyle interventions aimed at preventing diabetes. Pancreatic (PAT) and intermuscular (IMAAT) adipose tissues have also been identified as obesity-related fat depots that may contribute to metabolic alterations in childhood^8,9,10^ and adulthood.^9,11^

Lifestyle modification is the primary recommendation for childhood obesity treatment and prevention. Findings in the EFIGRO (Efectos de un Programa de Ejercicio Sobre la Grasa Hepatica en Niños con Sobrepeso u Obesidad) nonrandomized clinical trial^12^ showed that the addition of exercise to a family-based lifestyle intervention (ie, promotion of healthy lifestyle habits) program led to extra reductions in adiposity, insulin resistance, and hepatic steatosis in preadolescent children with overweight or obesity. Few studies compiled in a meta-analysis examined the effect of multicomponent programs on VAT in children with overweight or obesity, resulting in a medium effect of VAT reduction.^13^ However, no information is available on the effects of multicomponent intervention programs on a broad set of fat depots (ie, VAT, abdominal subcutaneous adipose tissue [ASAT], IMAAT, or PAT) using high-quality measuring methods, all of which have been associated with the risk of developing diabetes and cardiovascular morbidity and mortality. Moreover, there is no information regarding the potential mediating effect of a reduction in VAT on insulin resistance. The aims of the present study (performed within the EFIGRO framework) were (1) to compare the effects of a family-based lifestyle and psychoeducation program (designed according to current pediatric guidelines^14^) and those of the same program plus supervised exercise training on VAT, ASAT, IMAAT, and PAT in children aged 8 to 12 years with overweight or obesity and (2) to explore the mediating effect of VAT reduction on insulin resistance.

## Methods

### Study Design and Study Subjects

**Figure 1.**
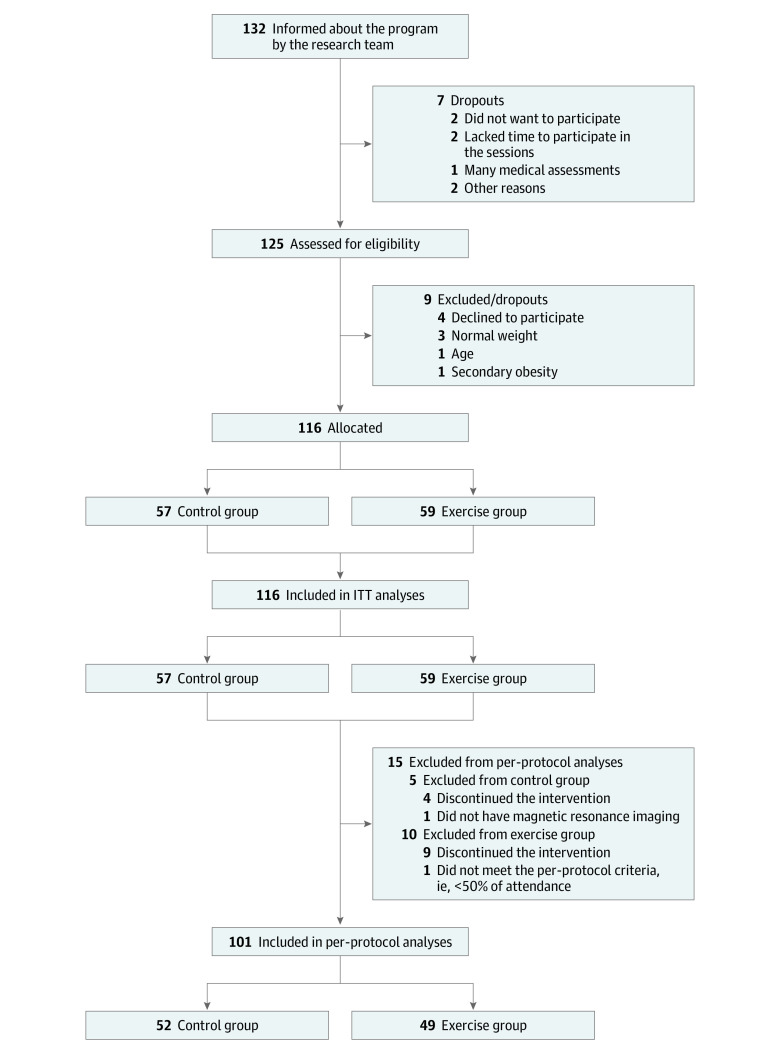
Flow Diagram of Participation, Data Collection, and Data Analysis The control group intervention consisted of 2 family-based lifestyle and psychoeducation sessions per month. The exercise group intervention was the same program plus 3 sessions of supervised exercise per week. ITT indicates intention to treat.

The EFIGRO project is a 2-group, parallel design clinical trial (1:1) designed to compare the changes in the percentage of hepatic fat between a family-based lifestyle and psychoeducation program (control group) with the same program plus a supervised exercise regimen (exercise group).^12,15^ Children and their families were recruited at the Pediatric Endocrinology Unit of the Hospital Universitario de Álava and at primary care clinics in Vitoria-Gasteiz, Spain. The study participants are all children aged 8 to 12 years with overweight or obesity^16^ but with no other medical condition that might limit their physical activity. A total of 116 children were allocated to either the control (n = 57) or exercise intervention (n = 59) groups. Data were collected between September 1, 2014, and June 30, 2017. Figure 1 shows the participation flowchart following the Consolidated Standards of Reporting Trials (CONSORT) guideline. In addition, the trial followed the Transparent Reporting of Evaluations With Nonrandomized Designs (TREND) reporting guideline. The study protocol was approved by the Clinical Research Ethics Committee of Euskadi. All participating children’s parents or legal guardians gave their written informed consent for their charges to participate, and the children themselves gave their assent. The trial protocol appears in Supplement 1.

### Power and Sample Size

Power calculation was based on the results of previous studies exploring visceral adiposity, insulin resistance, and total body fat percentage variables.^17,18,19^ Our study was powered to detect medium effect sizes (ie, Cohen *d* = 0.50), with an α error of 5% and a power of 80% with the inclusion of a minimum of 34 children in each group (68 participants were needed for sufficient power).^12,15^

### Intervention Program

#### Family-Based Lifestyle and Psychoeducation Program

The lifestyle program (two 45-minute sessions per month for 22 weeks) was based on the promotion of 3 main lifestyle habits: (1) healthy dietary habits, (2) physical activity, and (3) good sleep behavior. The objectives of the psychoeducation program (two 45-minute sessions per month for 22 weeks) were to provide the necessary skills for handling emotions and feelings and improving psychological well-being. Further details can be found in the eMethods in Supplement 2.

#### Exercise Program

The exercise program was based on the recommendations for physical activity in children, accounting for the requirements for aerobic and muscle-and bone-strengthening activities and games.^20^ The intervention consisted of 90 minutes of supervised and monitored exercise training 3 times/wk for 22 weeks. An extended description can be found in the eMethods in Supplement 2.

### Clinical Assessment and Follow-up

Clinical assessments were made at baseline and at week 22 of the intervention. Each assessment consisted of 3 separate visits to our research facilities, the first to record a medical history and collect fasting blood samples, the second for magnetic resonance imaging (MRI), and the third for anthropometric and fitness assessments.

For the present study, the primary outcome was the change in VAT (in terms of area and fat fraction); secondary outcomes included changes in ASAT (again in terms of area and fat fraction), IMAAT and PAT (fat fraction only), and insulin resistance. We measured VAT, ASAT, IMAAT, and PAT variables by MRI using a 1.5T system (Magnetom Avanto; Siemens). Semiautomatic software for fat segmentation was used to calculate VAT, ASAT, and IMAAT variables in 3 axial sections: L2 to L3, L3, and L4 to L5. An experienced researcher (A.S.) performed a coarse outlining of the abdominal viscera, excluding all muscular tissue. An active contours algorithm^21^ was then used to fine-tune the limits between the abdominal viscera and the internal border of the abdominal musculature. The K-means method^22,23^ was used for intensity value classification, and the values obtained were used as thresholds for classifying pixels as fat or muscle in the examined depots. To quantify PAT, 3-dimensional multiecho gradient sequences were analyzed using OsiriX software, version 9 (Pixmeo SARL) as previously reported.^24,25^ Three regions of interest were defined in the head, body, and tail of the pancreas. Percentage of PAT was calculated from the mean values of all 3 regions of interest. The same medical imagers performed all of these analyses and were blinded to treatment group. Further details regarding the MRI examinations can be found in the eMethods in Supplement 2. Insulin resistance was determined using the homeostasis model assessment (HOMA). Cardiorespiratory fitness was evaluated via the progressive maximal incremental treadmill test with respiratory gas analysis.^15,19^

### Statistical Analysis

Data were analyzed between May 1, 2019, and February 12, 2021. Descriptive characteristics of the study sample were recorded. Before analysis, outcomes were assessed for normality. Residual plots were inspected to assess heteroscedasticity. Extreme values (ie, below percentile 1 and above percentile 99) were winsorized to limit their influence.^26^

#### Main Association of the Control and Exercise Interventions With Abdominal Fat Depots

Differences in changes in VAT, ASAT, IMAAT, and PAT between the control and exercise groups were determined by analysis of covariance adjusting for baseline values, age, sex, and changes in height (when needed). The effect size, based on the Cohen *d* value, was also calculated and interpreted as small (Cohen *d* = 0.20-0.49), medium (Cohen *d* = 0.50-0.79), or large (Cohen *d* ≥ 0.80).^27^

To determine the number of participants who experienced a clinically meaningful change from baseline to post intervention, a within-individual change distribution was calculated. Participants were grouped as responders if the standardized effect size of the intervention was equal to or exceeded a Cohen *d* of 0.20 or as nonresponders if this value was less than 0.20. The χ^2^ test was used to compare the percentage of responders between groups.

All analyses were performed following per-protocol (ie, children and parents attended ≥50% of the family-based lifestyle and psychoeducation program sessions; in the exercise group, no minimum attendance of exercise sessions was required) and intention-to-treat principles. Additionally, as exploratory analyses, we performed the per-protocol analyses by excluding those children and parents who were not randomized (ie, 11 of 116). As sensitivity analyses, we performed the per-protocol analyses by additionally including a minimum of 50% attendance of the exercise sessions. For the intention-to-treat analyses, missing values at follow-up were obtained by multiple imputation using baseline and postintervention values and accounting for age, sex, and group. Imputations were performed by blocks based on the outcomes for VAT, ASAT, IMAAT, and PAT; cardiometabolic risk; and cardiorespiratory fitness.

#### Mediation of HOMA by VAT

To test whether the effects of the intervention on HOMA were mediated by changes in VAT, mediation analysis was performed after adjusting for baseline values, age, sex, and changes in height using the PROCESS macro procedure in SPSS, version 22 (IBM Corporation), with 10 000 bootstrap samples. The unstandardized (B) and standardized (β) regression coefficients are presented for the following equations: (1) regressing the mediator (ie, change in VAT) on the independent variable (ie, group), (2) regressing the dependent variable (ie, change in HOMA) on the independent variable (group), and (3) regressing the dependent variable on both the mediator and the independent variable. Indirect and total effect were also presented, and thus the percentage of the total effect was computed to explain how much of the total effect was explained by the mediation. The mediation analyses are reported in accordance with A Guideline for Reporting Mediation Analyses of Randomized Trials and Observational Studies.^28^ Similar tests were also made to access whether the effects of the exercise intervention on the change in VAT were mediated via changes in cardiorespiratory fitness. All analyses were performed using SPSS, version 22 (IBM Corporation) or R, version 4.0.3 (R Project for Statistical Computing). Statistical significance was set at 2-sided *P* < .05.

## Results

### Study Subjects

The mean (SD) age of the 116 participants included in the analysis was 10.6 (1.1) years. Sixty-two participants (53.4%) were girls, 54 (46.6%) were boys, and 67 (57.8%) presented with obesity, classified as age- and sex-specific cutoffs for body mass index. A total of 101 children (49 in the exercise group and 52 in the control group) successfully completed the trial (ie, met the criteria for per-protocol analyses). Participants who discontinued the intervention (n = 13), did not have MRI studies (n = 1), or did not meet the per-protocol criteria (n = 1) were included in the intention-to-treat analyses (Figure 1). The analysis presented in the following text is the per-protocol analysis; the intention-to-treat analysis is included in the eTable 4 and eFigures 3 to 5 in Supplement 2. eTable 1 in Supplement 2 shows the baseline characteristics of the children by intervention group.

### Adverse Events and Session Attendance

Exercise-related adverse events included knee and ankle pain (n = 2); no adverse events were recorded in the control group. The mean attendance rate to the family-based lifestyle and psychoeducation program was 85% for children and 83% for parents; mean adherence to the supervised training in the exercise groups was 72%.

### Primary Outcome: VAT

**Figure 2.**
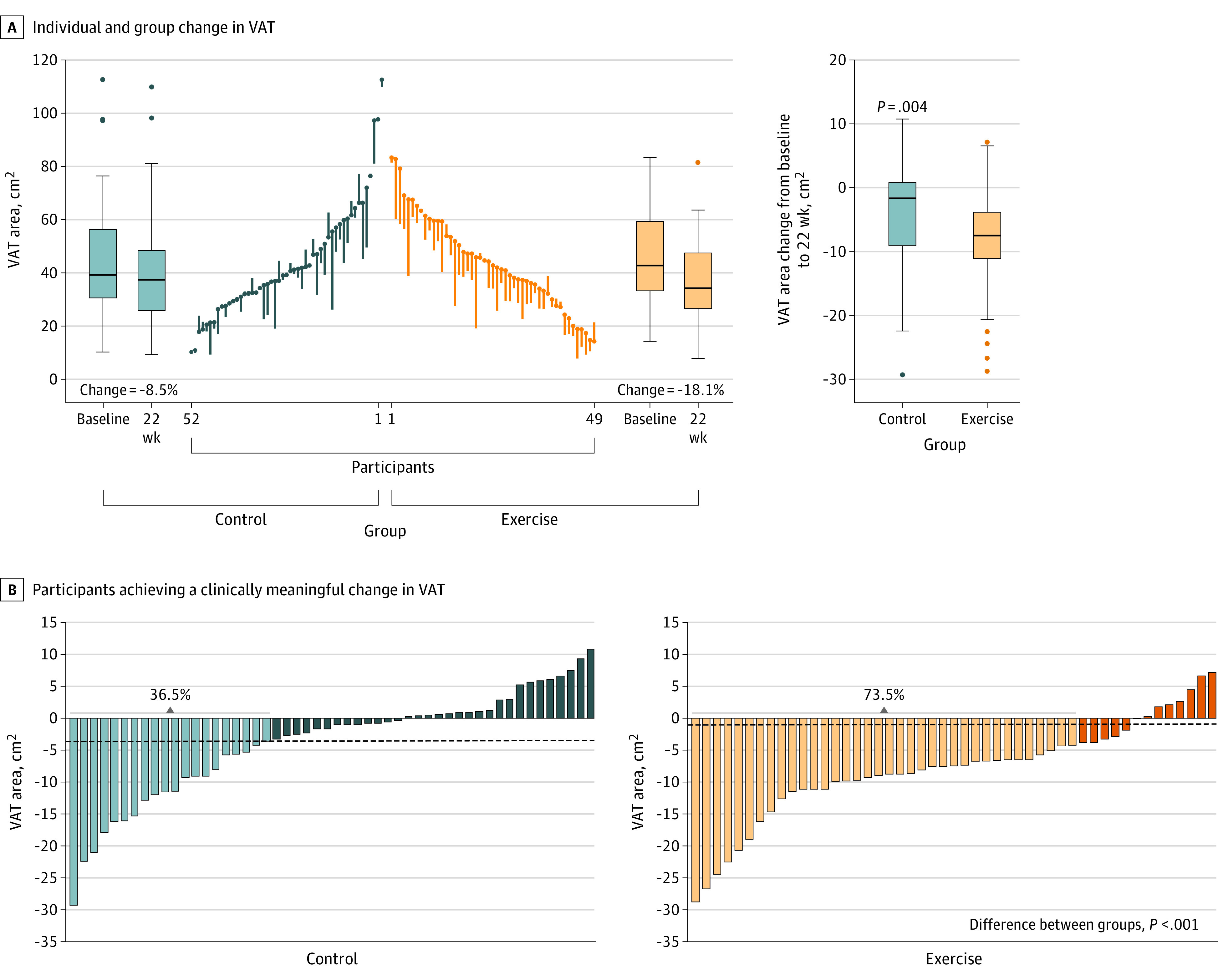
Changes in Abdominal Visceral Adipose Tissue (VAT) in the Control and Exercise Groups The control group intervention consisted of 2 family-based lifestyle and psychoeducation sessions per month. The exercise group intervention was the same program plus 3 sessions of supervised exercise per week. The ends of the boxes in the boxplots are located in the first and third quartiles; the black line in the middle illustrates the median. Whiskers extend to the upper and lower adjacent values. The location of the furthest point is within a distance of 1.5 × the interquartile range from the first and third quartiles. Data points above and below the error bars indicate the data of children outside the 95% CI range of the group. A, The parallel line plot contains 1 vertical line for each participant that extends from their baseline to their 22-week value. Descending lines indicate a reduction in VAT area. Pretest values are placed in ascending order for the control group and descending order for the exercise group. Changes were calculated as postintervention minus preintervention values. Analyses were adjusted for baseline values, age, sex, and changes in height. Data analyses were conducted under per-protocol conditions (ie, participants had to attend at least 50% of the family-based and psychoeducation program sessions; in the exercise group, no minimum attendance of exercise sessions was required). B, The lighter blue and orange bars represent those participants who experienced a clinically meaningful change (ie, responders) from baseline to post intervention (Cohen *d* ≥ 0.20). Darker blue and orange bars represent those who did not experience a clinically meaningful change (Cohen *d* < 0.20). The χ^2^ test was used to examine differences between groups (ie, control vs exercise).

Members of the exercise group experienced significantly greater reductions in VAT (area and fat fraction) compared with those in the control group from baseline to 22 weeks for area (Cohen *d* = 0.52 [*P* = .004]) and fat fraction (Cohen *d* = 0.78 [*P* < .001]) (Table and Figure 2A). The number of responders in terms of VAT area (73.5% vs 36.5%) (Figure 2B) and fat fraction reduction (81.6% vs 38.5%) (eFigure 1 in Supplement 2) was 40% higher in the exercise than in the control group (*P* < .001).

**Table.** Abdominal Visceral and Subcutaneous Adipose Tissue, Intermuscular Adipose Tissue, and Pancreatic Adipose Tissue at Baseline and After the Intervention in Per-Protocol Analysis

Abdominal adipose tissue	Mean (SD) result				Mean difference between groups (95% CI)^a^	*P* value^b^	Effect size, Cohen *d*^c^
	Control group (n = 52)		Exercise group (n = 49)				
	Preintervention	Postintervention	Preintervention	Postintervention			
Visceral adipose tissue							
Area, cm^2^	44.6 (21.5)	40.8 (21.4)	44.3 (17.7)	36.3 (16.3)	−4.2 (−7.5 to −1.0)	.004	0.52
Fat fraction, %	85.4 (2.7)	85.0 (3.0)	86.1 (2.6)	84.5 (2.8)	−1.2 (−1.8 to −0.6)	<.001	0.78
Subcutaneous adipose tissue							
Area, cm^2^	223.8 (70.8)	216.4 (79.6)	250.3 (72.8)	225.6 (79.6)	−17.2 (−27.7 to −6.7)	.001	0.65
Fat fraction, %	93.4 (1.3)	93.0 (1.9)	93.9 (1.0)	92.9 (1.6)	−0.5 (−0.9 to −0.1)	.002	0.36
Intermuscular abdominal adipose tissue							
Fat fraction, %	7.0 (1.0)	6.9 (1.1)	7.0 (0.8)	6.6 (0.7)	−0.3 (−0.5 to −0.01)	.02	0.42
Pancreatic adipose tissue^d^							
Fat fraction, %	3.2 (2.6)	2.9 (2.5)	3.2 (1.8)	2.6 (1.7)	−0.3 (−0.8 to 0.2)	.24	0.22

^a^ Calculated using the difference between groups (exercise minus control) of changes (postintervention minus preintervention).

^b^ Analyses were adjusted for baseline values, age, and sex except for visceral adipose tissue and subcutaneous adipose tissue area, for which adjustments were also made for changes in height.

^c^ Interpreted as small (Cohen *d* = 0.20), medium (Cohen *d* = 0.50), or large (Cohen *d* = 0.80).

^d^ The sample size was reduced for the control group (n = 50) and exercise group (n = 48).

Separate analyses of the 3 axial sections are shown in eTable 2 in Supplement 2. Sensitivity analyses using the per-protocol principle and additionally including a minimum of 50% attendance of the exercise sessions (97 participants [45 in the exercise group]) showed practically identical findings to the per-protocol principle applied (ie, no minimum attendance of exercise was required) (eTable 3 and eFigure 2 in Supplement 2). The intention-to-treat analyses returned similar results to the per-protocol analyses (eTable 4 and eFigure 3 in Supplement 2). Likewise, when we repeated the analyses (ie, exploratory analysis) excluding those children and parents who were not randomized (ie, 11 of 116), the findings were similar to those of the primary analysis (eTable 5 in Supplement 2)

### Secondary Outcomes: ASAT, IMAAT, and PAT

**Figure 3.**
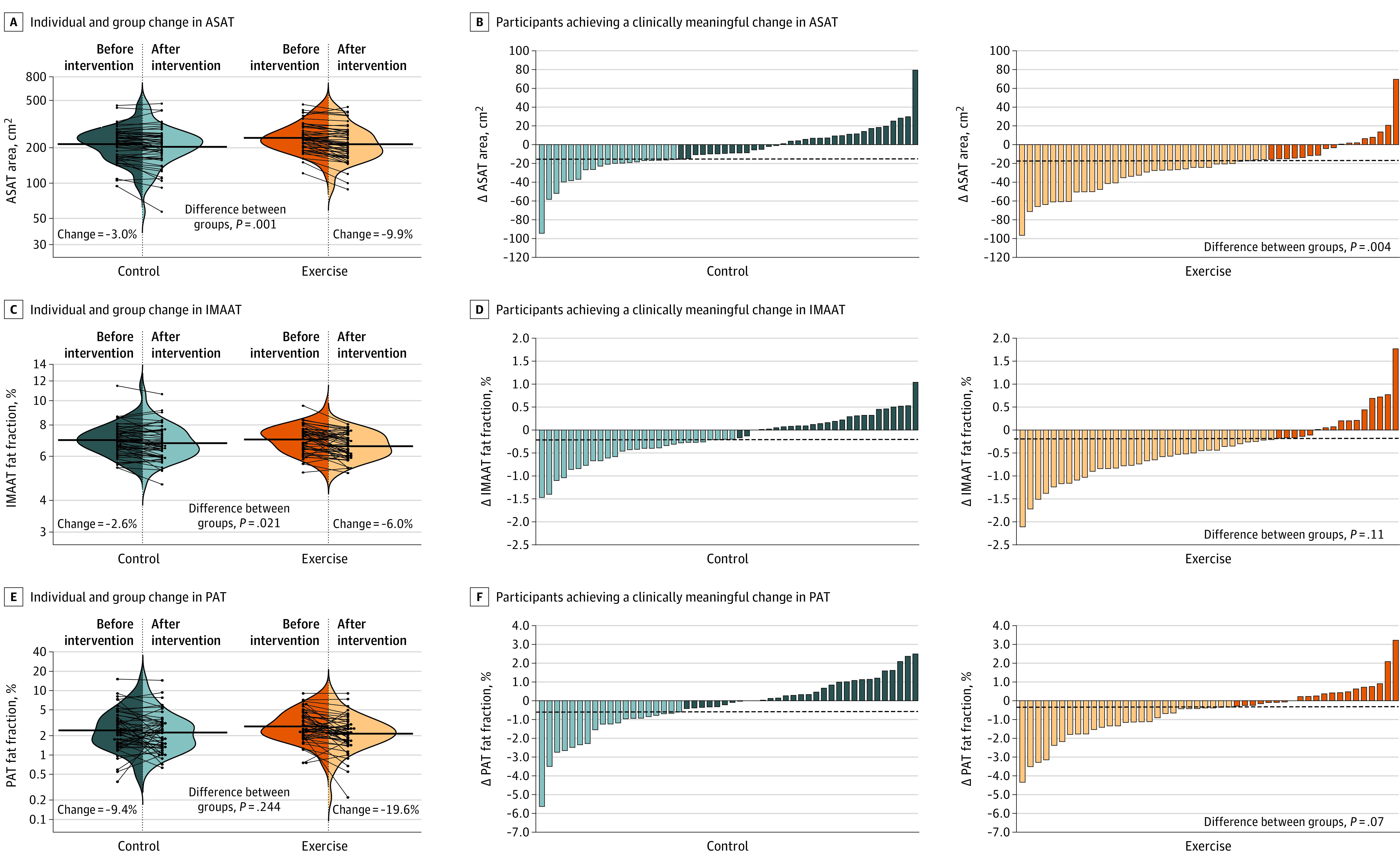
Changes in Abdominal Subcutaneous Adipose Tissue (ASAT), Intermuscular Abdominal Tissue (IMAAT), and Pancreatic Adipose Tissue (PAT) The control group intervention consisted of 2 sessions of family-based lifestyle and psychoeducation per month. The exercise group intervention was the same program plus 3 sessions of supervised exercise per week. Changes were calculated as postintervention minus preintervention values. Analyses were adjusted for baseline values, age, and sex. For difference in ASAT area, an additional adjustment was made for changes in height. Data analyses were conducted under the per-protocol principle (ie, subjects had to attend ≥50% of the family-based and psychoeducation program sessions; in the exercise group, no minimum attendance of exercise sessions was required). The lighter blue and orange bars represent those participants who experienced a clinically meaningful change (ie, responders) from baseline to post intervention (Cohen *d* ≥ 0.20). The darker blue and orange bars represent those participants who did not experience a clinically meaningful change (Cohen *d* < 0.20). Differences between the control and exercise groups were examined using the χ^2^ test.

The reductions in ASAT area (Cohen *d* = 0.65 [*P* = .001]) and fat fraction (Cohen *d* = 0.36 [*P* = .002]) (Table) and IMAAT fat fraction (Cohen *d* = 0.42 [*P* = .02]) (Table) were significantly greater for the exercise group than for the control group (Table and Figure 3A and C). The number of responders with respect to ASAT area was also 29% higher in the exercise group (65.3% vs 36.5%; *P* = .004), whereas no significant difference was seen with respect to IMAAT fat fraction (although reduction was 15% greater in the exercise group) (Figure 3B and D). Separate analyses of the 3 axial sections are shown in eTable 2 in Supplement 2.

For PAT fat fraction, no significant difference was seen between the 2 groups (Cohen *d* = 0.22 [*P* = .24]) (Table and Figure 3E). The number of responders for PAT was 18% higher in the exercise than in the control group (*P* = .07) (Figure 3F). The intention-to-treat analyses returned results similar to those of the per-protocol analyses (eTable 4 and eFigure 4 in Supplement 2). As an exploratory analysis, after excluding those participants who were not randomized, the results were similar (eTable 5 in Supplement 2).

### Mediation of HOMA by VAT

**Figure 4.**
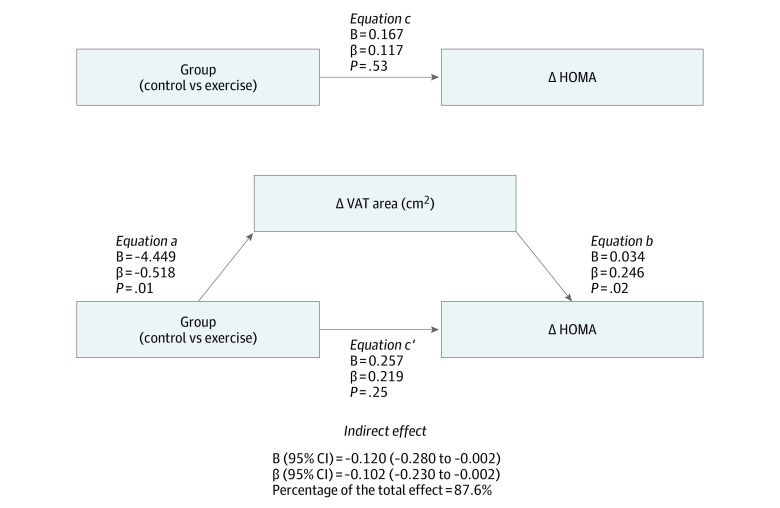
Mediation Model to Determine Whether Changes in Visceral Adipose Tissue (VAT) Area Mediated Changes in Insulin Resistance Data presented are for per-protocol analysis. Adjustments were made for baseline values, age, sex, and changes in height. Delta (∆) expresses the postintervention outcome with respect to baseline. HOMA indicates homeostasis model assessment.

Figure 4 shows the mediation model of how changes in VAT area affected HOMA in both groups. Changes in VAT area explained 87.6% of the improvements in HOMA recorded (both groups taken together) (indirect effect: β = −0.102 [95% CI, −0.230 to −0.002]) (Figure 4). The intention-to-treat analyses returned similar results (eFigure 5 in Supplement 2). When we excluded those participants who were not randomized (ie, exploratory analysis), the findings were similar to those for the primary analysis (eFigure 6 in Supplement 2). Finally, the change in cardiorespiratory fitness could mediate the change in VAT area (indirect effect: β = −0.126 [95% CI, −0.284 to −0.014]), explaining 26% of the total effect.

## Discussion

The present results show that the addition of exercise training to a 22-week family-based lifestyle and psychoeducation program is associated with greater, and indeed clinically meaningful, reductions in abdominal VAT, ASAT, and IMAAT. In addition, the reduction in VAT area could mediate the improvement recorded in insulin sensitivity, highlighting the importance of targeting childhood obesity therapies for this fat depot.

Current pediatric guidelines^14^ based on quality of evidence underline (1) the effectiveness of multicomponent weight management interventions (ie, diet and nutrition, physical activity, and behavioral components) and (2) the need for family participation in multicomponent pediatric weight management interventions. However, these guidelines provide no specific instructions or methodological information on how interventions should be implemented. Moreover, they focus on reducing body mass index or waist circumference. The present trial goes beyond this format by focusing on a broad set of fat depots, by using high-quality measuring methods, and by detailing an effective intervention procedure. Treating and reducing obesity in childhood is important because it has been recently shown that the prepubertal period (ie, younger than 13 years) is critical in terms of reducing the risk of developing type 2 diabetes.^29^

Excess VAT has been associated with many metabolic abnormalities and is a strong predictive factor associated with morbidity and all-cause mortality in adults.^30^ Indeed, excess VAT is more detrimental than excess body weight.^31^ Few studies, however, have examined the effect of multicomponent programs on VAT in children with overweight or obesity.^13^ The present work reveals a medium-size effect (0.5 SD) of the multicomponent intervention with exercise on VAT reduction. Interestingly, the number of responders in the exercise group was double that recorded for the control group (73.5% vs 36.5%). These findings agree with the results of a published meta-analysis^13^ and those of more recent literature,^32^ which indicate combined diet and exercise interventions, as well as exercise-only interventions, may both result in a significant reduction in VAT in children and adolescents with overweight or obesity^13^ (in the above meta-analysis, the overall weighted mean effect size ranged from −0.55 to −0.85, dropping to −0.49 when only those studies with a control group were included).^13^ However, in contrast with the present findings, Dias et al^33^ reported neither 12 weeks of high-intensity interval training nor moderate-intensity continuous training to be effective in reducing VAT in 65 children with obesity.

With respect to ASAT, a robust reduction (10%) and a medium to large effect size (0.7 SD) was seen for the exercise group. Similar to the findings for VAT, the exercise group had almost double the percentage of responders (65.3% vs 36.5% for the control group). This outcome agrees with the results of most previous randomized clinical trials in which a greater reduction in ASAT was seen for exercise-including interventions compared with controls in both children and adolescents.^19,34,35^ Other studies have reported no evidence of any effect,^33,36^ but the small sample sizes (44-65 participants), the differences in participant characteristics, and the type of exercise program followed in these studies might well explain these discrepancies.

The present exercise program had a medium effect size for IMAAT fat fraction (0.4 SD). Unfortunately, the literature contains no information on preadolescent children that might be used in comparisons. However, the present findings do agree with those of Lee et al,^37^ who found a reduction in IMAAT in adolescent participants with overweight or obesity who followed a combined resistance and aerobic exercise program. In any event, the limited information available broadly agrees with the present findings, suggesting that a multicomponent intervention program that includes exercise can reduce IMAAT effectively, highlighting the usefulness of including exercise in treatments for overweight or obesity.

For PAT, no significant difference was seen between the 2 intervention groups. This result is supported by the findings of 2 randomized clinical trials that examined pancreatic fat reduction in adults.^38,39^ To the best of our knowledge, the present work is the first to compare the outcomes of a lifestyle and a multicomponent intervention program on PAT in children.

Insulin resistance is a problem that might be reversed by substantial VAT loss, and the present work highlights that exercise-induced reduction in VAT might mediate a reduction in insulin resistance. This outcome should help protect against the development of type 2 diabetes.

Our findings suggest that the addition of exercise combining aerobic and resistance training with family-based lifestyle programs for children with overweight or obesity promotes the reduction of a broad set of harmful ectopic fat depots and therefore reduces the risk of developing type 2 diabetes. These findings emphasize the importance of recommending the inclusion of exercise as a cornerstone of public health strategies for treating childhood obesity and preventing the development of type 2 diabetes.

### Strengths and Limitations 

The study has several strengths, including assessment of the abdominal fat depots by MRI. The assessing researchers were entirely blinded to the intervention group; therefore, the outcome of interest in this study (ie, MRI findings) was analyzed unbiased.

This present study also has several limitations. First, randomization was not entirely strict; not all children and parents (11 of 116 participants) could meet the time demands of the exercise group but could meet those of the control group, and they were therefore assigned accordingly. For ethical purposes, we could not exclude them from the trial because we were aware of its benefits in this population. However, given that participant characteristics were comparable in both groups at baseline and that between-group analyses were adjusted for baseline values, we believe it is unlikely that this factor would modify the study’s conclusions. Nevertheless, we repeated all of the analyses excluding those children and parents who were not randomized, and the findings obtained were similar to those of the primary analysis (eTable 5 and eFigure 6 in Supplement 2). Second, results of the mediation analyses should be interpreted with caution because both the proposed mediator and the outcome were measured at the same time.

## Conclusions

A 22-week family-based lifestyle and psychoeducation intervention reduced abdominal fat in children with overweight or obesity, but the addition of exercise training promoted even greater—and clinically meaningful—reductions in VAT, ASAT, and IMAAT. The reduction in VAT might mediate the improvement in insulin sensitivity. These results highlight the importance of including exercise training in lifestyle therapies for childhood obesity; this component promotes reductions in the size of harmful ectopic fat depots and therefore reduces the risk of developing type 2 diabetes. Further research is needed to assess the long-term clinical outcomes of such interventions.

## Notes

Supplement 1.
Trial Protocol

Supplement 2. **eMethods.** Family-Based Lifestyle and Psychoeducation Program, Exercise Intervention Program, and Data Analysis
eReferences
**eTable 1.** Subject Characteristics **eTable 2.** Abdominal Visceral, Subcutaneous, and Intermuscular Adipose Tissue From 3 Axial Sections at Baseline and After the Intervention (Per-Protocol Analysis) **eTable 3.** Abdominal Visceral and Subcutaneous Adipose Tissue, Intermuscular Adipose Tissue, and Pancreatic Adipose Tissue at Baseline and After the Intervention (Per-Protocol Analysis Additionally Including a Minimum of 50% of Attendance of the Exercise Sessions) **eTable 4.** Abdominal Visceral and Subcutaneous Adipose Tissue, Intermuscular Adipose Tissue, and Pancreatic Adipose Tissue at Baseline and After the Intervention (Intention-to-Treat Analyses) **eTable 5.** Abdominal Visceral and Subcutaneous Adipose Tissue, Intermuscular Adipose Tissue, and Pancreatic Adipose Tissue at Baseline and After the Intervention (Per-Protocol Analysis Using Only Those Children and Parents Who Were Randomized) **eFigure 1.** Changes in Abdominal Visceral Adipose Tissue and Abdominal Subcutaneous Adipose Tissue in Participants in the Lifestyle and Psychoeducational Program (Control Group) and the Same Program Plus Supervised Exercise Training (Exercise Group) **eFigure 2.** Mediation Model to Determine Whether Changes in VAT Area Mediated Changes in Insulin Resistance (HOMA) **eFigure 3.** Changes in Abdominal Visceral Adipose Tissue in Participants in the Lifestyle and Psychoeducation Program (Control Group) and in the Same Program Plus Supervised Exercise Training (Exercise Group) in Intention-to-Treat Analyses **eFigure 4.** Changes in Abdominal Subcutaneous Adipose Tissue, Intermuscular Abdominal, and Pancreatic Fat Fraction in Participants in the Lifestyle and Psychoeducation Program (Control Group) and in the Same Program Plus Supervised Exercise Training (Exercise Group) in Intention-to-Treat Analyses **eFigure 5.** Mediation Model to Determine Whether Changes in VAT Area Mediated Changes in Insulin Resistance (HOMA) in Intention-to-Treat Analyses **eFigure 6.** Mediation Model to Determine Whether Changes in VAT Area Mediated Changes in Insulin Resistance (HOMA) in Sensitivity Analysis Using Only Those Children Who Were Randomized

Supplement 3.
Data Sharing Statement

## References

[1] Fox CS, Massaro JM, Hoffmann U, . Abdominal visceral and subcutaneous adipose tissue compartments: association with metabolic risk factors in the Framingham Heart Study. Circulation. 2007;116(1):39-48. doi:10.1161/CIRCULATIONAHA.106.675355 17576866

[2] Wajchenberg BL. Subcutaneous and visceral adipose tissue: their relation to the metabolic syndrome. Endocr Rev. 2000;21(6):697-738. doi:10.1210/edrv.21.6.0415 11133069

[3] Després J-P, Lemieux I. Abdominal obesity and metabolic syndrome. Nature. 2006;444(7121):881-887. doi:10.1038/nature05488 17167477

[4] Kuk JL, Katzmarzyk PT, Nichaman MZ, Church TS, Blair SN, Ross R. Visceral fat is an independent predictor of all-cause mortality in men. Obesity (Silver Spring). 2006;14(2):336-341. doi:10.1038/oby.2006.43 16571861

[5] Katzmarzyk PT, Mire E, Bouchard C. Abdominal obesity and mortality: the Pennington Center Longitudinal Study. Nutr Diabetes. 2012;2(8):e42. doi:10.1038/nutd.2012.15 23168527 PMC3432185

[6] McNeely MJ, Shofer JB, Leonetti DL, Fujimoto WY, Boyko EJ. Associations among visceral fat, all-cause mortality, and obesity-related mortality in Japanese Americans. Diabetes Care. 2012;35(2):296-298. doi:10.2337/dc11-1193 22190675 PMC3263911

[7] Maffeis C, Morandi A. Body composition and insulin resistance in children. Eur J Clin Nutr. 2018;72(9):1239-1245. doi:10.1038/s41430-018-0239-2 30185840

[8] Cohen M, Syme C, Deforest M, . Ectopic fat in youth: the contribution of hepatic and pancreatic fat to metabolic disturbances. Obesity (Silver Spring). 2014;22(5):1280-1286. doi:10.1002/oby.20674 24402863

[9] Lê KA, Ventura EE, Fisher JQ, . Ethnic differences in pancreatic fat accumulation and its relationship with other fat depots and inflammatory markers. Diabetes Care. 2011;34(2):485-490. doi:10.2337/dc10-0760 21270204 PMC3024373

[10] Staaf J, Labmayr V, Paulmichl K, . Pancreatic fat is associated with metabolic syndrome and visceral fat but not beta-cell function or body mass index in pediatric obesity. Pancreas. 2017;46(3):358-365. doi:10.1097/MPA.0000000000000771 27941426 PMC5312728

[11] Nachit M, Kwanten WJ, Thissen JP, . Muscle fat content is strongly associated with NASH: a longitudinal study in patients with morbid obesity. J Hepatol. 2021;75(2):292-301. doi:10.1016/j.jhep.2021.02.037 33865909

[12] Labayen I, Medrano M, Arenaza L, . Effects of exercise in addition to a family-based lifestyle intervention program on hepatic fat in children with overweight. Diabetes Care. 2020;43(2):306-313. doi:10.2337/dc19-0351 31227585

[13] Vissers D, Hens W, Hansen D, Taeymans J. The effect of diet or exercise on visceral adipose tissue in overweight youth. Med Sci Sports Exerc. 2016;48(7):1415-1424. doi:10.1249/MSS.0000000000000888 27314412

[14] Styne DM, Arslanian SA, Connor EL, . Pediatric obesity-assessment, treatment, and prevention: an Endocrine Society clinical practice guideline. J Clin Endocrinol Metab. 2017;102(3):709-757. doi:10.1210/jc.2016-2573 28359099 PMC6283429

[15] Medrano M, Maiz E, Maldonado-Martín S, . The effect of a multidisciplinary intervention program on hepatic adiposity in overweight-obese children: protocol of the EFIGRO study. Contemp Clin Trials. 2015;45(pt B):346-355. doi:10.1016/j.cct.2015.09.017 26408054

[16] Cole TJ, Lobstein T. Extended international (IOTF) body mass index cut-offs for thinness, overweight and obesity. Pediatr Obes. 2012;7(4):284-294. doi:10.1111/j.2047-6310.2012.00064.x 22715120

[17] Owens S, Gutin B, Allison J, . Effect of physical training on total and visceral fat in obese children. Med Sci Sports Exerc. 1999;31(1):143-148. doi:10.1097/00005768-199901000-00022 9927022

[18] Ferguson MA, Gutin B, Le NA, . Effects of exercise training and its cessation on components of the insulin resistance syndrome in obese children. Int J Obes Relat Metab Disord. 1999;23(8):889-895. doi:10.1038/sj.ijo.0800968 10490792

[19] Davis CL, Pollock NK, Waller JL, . Exercise dose and diabetes risk in overweight and obese children: a randomized controlled trial. JAMA. 2012;308(11):1103-1112. doi:10.1001/2012.jama.10762 22990269 PMC3487697

[20] Bull FC, Al-Ansari SS, Biddle S, . World Health Organization 2020 guidelines on physical activity and sedentary behaviour. Br J Sports Med. 2020;54(24):1451-1462. doi:10.1136/bjsports-2020-102955 33239350 PMC7719906

[21] Kass M, Witkin A, Terzopoulos D. Snakes: active contour models. Int J Comput Vis. 1988;1(4):321-331. doi:10.1007/BF00133570

[22] Lloyd S. Least squares quantization in PCM. IEEE Trans Inf Theory. 1982;28(2):129-137. doi:10.1109/TIT.1982.1056489

[23] Forgy E. Cluster analysis of multivariate data: efficiency versus interpretability of classifications. Biometrics. 1965;21(3):768-769.

[24] Chen Y, Long L, Jiang Z, Zhang L, Zhong D, Huang X. Quantification of pancreatic proton density fat fraction in diabetic pigs using MR imaging and IDEAL-IQ sequence. BMC Med Imaging. 2019;19(1):38. doi:10.1186/s12880-019-0336-2 31088378 PMC6515681

[25] Covarrubias Y, Fowler KJ, Mamidipalli A, . Pilot study on longitudinal change in pancreatic proton density fat fraction during a weight-loss surgery program in adults with obesity. J Magn Reson Imaging. 2019;50(4):1092-1102. doi:10.1002/jmri.26671 30701611 PMC6667307

[26] Sink KM, Espeland MA, Castro CM, ; LIFE Study Investigators. Effect of a 24-month physical activity intervention vs health education on cognitive outcomes in sedentary older adults: the LIFE randomized trial. JAMA. 2015;314(8):781-790. doi:10.1001/jama.2015.9617 26305648 PMC4698980

[27] Cohen J. A power primer. Psychol Bull. 1992;112(1):155-159. doi:10.1037/0033-2909.112.1.155 19565683

[28] Lee H, Cashin AG, Lamb SE, ; AGReMA group. A guideline for reporting mediation analyses of randomized trials and observational studies: the AGReMA Statement. JAMA. 2021;326(11):1045-1056. doi:10.1001/jama.2021.14075 34546296 PMC8974292

[29] Bjerregaard LG, Jensen BW, Ängquist L, Osler M, Sørensen TIA, Baker JL. Change in overweight from childhood to early adulthood and risk of type 2 diabetes. N Engl J Med. 2018;378(14):1302-1312. doi:10.1056/NEJMoa1713231 29617589

[30] Brown JC, Harhay MO, Harhay MN. Visceral adipose tissue dysfunction and mortality among a population-based sample of males and females. Diabetes Metab. 2016;42(5):382-385. doi:10.1016/j.diabet.2016.05.001 27283873 PMC5140761

[31] Chang YH, Yang HY, Shun SC. Effect of exercise intervention dosage on reducing visceral adipose tissue: a systematic review and network meta-analysis of randomized controlled trials. Int J Obes (Lond). 2021;45(5):982-997. doi:10.1038/s41366-021-00767-9 33558643

[32] Lee S, Libman I, Hughan KS, . Effects of exercise modality on body composition and cardiovascular disease risk factors in adolescents with obesity: a randomized clinical trial. Appl Physiol Nutr Metab. 2020;45(12):1377-1386. doi:10.1139/apnm-2019-0993 32674587

[33] Dias KA, Ingul CB, Tjønna AE, . Effect of high-intensity interval training on fitness, fat mass and cardiometabolic biomarkers in children with obesity: a randomised controlled trial. Sports Med. 2018;48(3):733-746. doi:10.1007/s40279-017-0777-0 28853029

[34] Davis JN, Gyllenhammer LE, Vanni AA, . Startup circuit training program reduces metabolic risk in Latino adolescents. Med Sci Sports Exerc. 2011;43(11):2195-2203. doi:10.1249/MSS.0b013e31821f5d4e 21502883 PMC3480316

[35] Lee S, Bacha F, Hannon T, Kuk JL, Boesch C, Arslanian S. Effects of aerobic versus resistance exercise without caloric restriction on abdominal fat, intrahepatic lipid, and insulin sensitivity in obese adolescent boys: a randomized, controlled trial. Diabetes. 2012;61(11):2787-2795. doi:10.2337/db12-0214 22751691 PMC3478522

[36] Lee S, Deldin AR, White D, . Aerobic exercise but not resistance exercise reduces intrahepatic lipid content and visceral fat and improves insulin sensitivity in obese adolescent girls: a randomized controlled trial. Am J Physiol Endocrinol Metab. 2013;305(10):E1222-E1229. doi:10.1152/ajpendo.00285.2013 24045865 PMC3840217

[37] Lee S, Libman I, Hughan K, . Effects of exercise modality on insulin resistance and ectopic fat in adolescents with overweight and obesity: a randomized clinical trial. J Pediatr. 2019;206:91-98.e1. doi:10.1016/j.jpeds.2018.10.059 30554789 PMC7193538

[38] Tene L, Shelef I, Schwarzfuchs D, . The effect of long-term weight-loss intervention strategies on the dynamics of pancreatic-fat and morphology: an MRI RCT study. Clin Nutr ESPEN. 2018;24:82-89. doi:10.1016/j.clnesp.2018.01.008 29576369

[39] Heiskanen MA, Motiani KK, Mari A, . Exercise training decreases pancreatic fat content and improves beta cell function regardless of baseline glucose tolerance: a randomised controlled trial. Diabetologia. 2018;61(8):1817-1828. doi:10.1007/s00125-018-4627-x 29717337 PMC6061150

