# Supplementary material for: Effects of a Family-Based Lifestyle Intervention Plus Supervised Exercise Training on Abdominal Fat Depots in Children With Overweight or Obesity: A Secondary Analysis of a Nonrandomized Clinical Trial
Source: JAMA Netw Open. 2022 Nov 28;5(11):e2243864. doi: 10.1001/jamanetworkopen.2022.43864 (PMC9706365; doi:10.1001/jamanetworkopen.2022.43864)
Supplement: Supplement 1. — Trial Protocol [file jamanetwopen-e2243864-s001.pdf]

CLINICAL TRIAL PROTOCOL

The Effect of Exercise on Hepatic Fat in Overweight Children; the EFIGRO Study

**Brief title**
Effect of Exercise on Hepatic Fat in Overweight Children
**Organization's Unique Protocol ID**
NCT02258126 / PI13/01335
**Study Start**
November 2014
**Study completion**
November 2017
**Sponsor**
University of the Basque Country (UPV/EHU)
**Principal investigator**
Idoia Labayen, Associate Professor.
**Principal collaborators**
University Hospital Alava
**Human subjects review**
PI2014045

Table of contents

PROTOCOL SUMMARY ... 3
BACKGROUND ... 5
Hypothesis ... 7
Objectives ... 7
Methods ... 8
Study design ... 8
Participants and selection criteria ... 8
Sample size calculation ... 9
Randomization ... 10
Exercise training intervention ... 10
Rationale and design of the healthy lifestyle education and psychoeducation interventions ... 11
Aims and content of the psychoeducation intervention ... 14
Participant retention and addressing compliance and adherence ... 15
Measurements ... 16
Primary outcome ... 17
Secondary outcomes measures ... 18
Assessment of side effects ... 26
Data analysis plan ... 27
Discussion ... 28
ACKNOWLEDGMENTS ... 29
REFERENCES ... 30
PARTICIPANTS INFORMATIVE SHEET AND INFORMED CONSENT ... 40
ETHIC COMMITTEE APPROVAL ... 46

PROTOCOL SUMMARY

Non-alcoholic fatty liver disease is the most frequent liver abnormality observed in overweight or obese children and is strongly associated with metabolic syndrome and insulin resistance. Therefore, the aims of this project are: (i) To evaluate the effect of a 22 weeks multidisciplinary intervention program on hepatic fat fraction in overweight or obese children and (ii) To examine the effect of the intervention on cardiometabolic risk factors, self-esteem and well-being. A total of 160 children, 9-11 years, will be recruited by paediatricians and randomly assigned to control (N=80) or intervention (N=80) groups. The control group will receive a family-based lifestyle and psychoeducation program (2 days/month), while the intervention group will attend the same lifestyle education and psychoeducation program plus the exercise program (3 days/week). The duration of training sessions will be 90min of exercise, including warm-up, moderate to vigorous aerobic activities, and strength exercises. The primary outcome is the change in hepatic fat fraction (magnetic resonance imaging, MRI). Secondary outcomes include cardiometabolic risk factors such as total and adiposity (dual X-ray absorptiometry), visceral adiposity (MRI), functional peak aerobic capacity (cardiopulmonary exercise testing), blood pressure, muscular fitness, speed agility, and fasting blood insulin, glucose, C-reactive protein, alanine aminotransferase, aspartate aminotransferase, gamma glutamyltransferase, lipid profile and psychological measurements (questionnaires). All the measurements will be evaluated at baseline prior to randomization and after the intervention. This study will provide insight in the efficacy of a multidisciplinary intervention program including healthy lifestyle education, psychoeducation and supervised exercise to reduce hepatic fat and cardiometabolic risk in overweight children.

Trial registration: NCT02258126

Keywords: fatty liver, obesity, children, exercise, metabolic syndrome

LIST OF ABREVIATIONS

ALT: alanine aminotransferase; AST: aspartate aminotransferase; BMI: body mass index; CRP: C-reactive protein; Gamma-GT: gamma glutamyltransferase; LDL: low density lipoprotein cholesterol; NAFLD: non-alcoholic fatty liver disease; TG: triglycerides; VCO_2: carbon dioxide production; VO_{2max}: maximum oxygen consumption.

BACKGROUND

Childhood obesity is a global epidemic and a major public health problem (1). In Europe, as well as in other developed countries, the prevalence of paediatric overweight is ranging from 20% to 40%. Spain, together with other countries surrounding the Mediterranean Sea, shows the highest rates of this well-recognized public health problem (2).

There has also been a concomitant rise of health complications associated with excess adiposity including dyslipidemia, hypertension, abnormal glucose tolerance, reduced quality of life and psychological distresses (3). The rise in the prevalence rates of overweight in childhood which occurred in the last two decades may explain the emergence of non-alcoholic fatty liver disease (NAFLD) as the leading cause of chronic liver disease in developed nations (4, 5). The real prevalence of pediatric NAFLD is still not defined because (i) to date only a few population studies have been conducted in children and (ii) because of the different screening/diagnosis method used. Moreover, sex and ethnic differences have also been reported (6). Likewise, previous studies estimated that up to 70-90% of obese youths aged 2 to 19 years were affected by NAFLD (7).A children's autopsy study conducted to estimate the prevalence of NAFLD in the United States observed that 38% of obese children were affected by NAFLD (8). The condition of NAFLD in children is still under-diagnosed because paediatric patients are often asymptomatic, and liver enzyme levels are usually mildly elevated in NAFLD in children and may remain normal, even with biopsy proven hepatic steatosis (9, 10).

Paediatric NAFLD is strongly associated with several factors of metabolic syndrome such as insulin resistance, abdominal adiposity, hypertension and dyslipidaemia (5, 11, 12). Therefore, NAFLD has been considered the hepatic manifestation of metabolic syndrome (13). Strategies to reduce or prevent NAFLD in

overweight children have the potential to improve overall cardiometabolic risk and reduce the risk to develop cardiovascular disease, type 2 diabetes and liver dysfunction (14).

There is evidence that sedentary lifestyle and unhealthy diets are driving the obesity epidemic and its co-morbidities in children. In turn, lifestyle interventions including family-based nutritional education, psychoeducation, and physical activity are recognized as key factors in the obesity treatment or prevention in paediatric population (15-18). Moreover, interventions including exercise have the potential to improve psychological aspects such as self-esteem and well-being in overweight children (19).

Currently, there are no dietetic and physical activity guidelines for the management of NAFLD in children (20). In adults, weight loss is able to attenuate or even reverse the course of NAFLD (21). However, energy restriction programs may lead to undesirable loss of lean mass and could compromise healthy growth and development in children. Previous studies in children have shown that exercise reduces cardiometabolic risk factors such as visceral adiposity and insulin resistance (22), low density lipoprotein cholesterol (LDL) and triglycerides (TG) levels (23) or blood pressure (24). The majority of the exercise-based intervention studies focused on hepatic steatatosis have been conducted in adults. As far as we are aware, there are only three previous studies in adolescents examining the effect of supervised exercise without caloric restriction on hepatic fat, and no one in pre-pubertal children (25-27). Given that: (i) multidisciplinary approaches to change or prevent unhealthy lifestyle targeting on modifiable factors are critical to improve obesity related disorders, especially in the long-term, and (ii) exercise has been associated with improvements in fatty liver, we propose a multidisciplinary intervention program including family-based

nutritional education, psychological education, and supervised aerobic and strength activities to reduce hepatic fat accumulation in overweight pre-pubertal children.

Hypothesis

Aerobic and strength training together improves insulin sensitivity and reduces visceral adiposity in overweight children. Given that hepatic fat accumulation is strongly linked to visceral adiposity and insulin resistance, our hypothesis is that a multidisciplinary intervention program including supervised aerobic and strength training will reduce hepatic fat fraction in overweight children.

Objectives

The primary objective of the EFIGRO study is to investigate the additional effect of aerobic and strength training on hepatic fat fraction in overweight and obese children participating in a 22 weeks family-based healthy lifestyle education and psychoeducation program.

Secondary objectives of the study are (i) to examine the additional effect of aerobic and strength training on cardiometabolic risk in overweight and obese children participating in a 22 weeks family-based healthy lifestyle education and psychoeducation program and (ii) to evaluate the additional effect of the exercise intervention in self-esteem and well-being of overweight and obese children.

Methods

Study design

The present study is a RCT (ClinicalTrials.gov ID: NCT02258126). All parents or guardians sign an informed written consent and all the children give their assent before being enrolled in the study. The study protocol has been approved by the Ethic Committee of Clinical Investigation of Euskadi (PI2014045). Since growing children are increasing in body size and undergoing developmental changes, a control group is necessary to detect changes due to training. Therefore, we compare relative changes between control and exercise groups. After baseline measurements, children are randomly allocated to the control or exercise group. Participants are followed for 22 weeks. All families enrolled in the study participate in a lifestyle education and psychoeducation programs. The intervention is performed in waves so that each wave has from 15 to 20 participants. Follow-up examinations are performed in the same settings, with the same instruments and by the same investigators as in the baseline measurements.

Participants and selection criteria

Participants are recruited from the Paediatric Endocrinology Unit of the University Hospital of Araba (HUA) (Vitoria-Gasteiz, Spain). Moreover, paediatricians in Vitoria-Gasteiz are invited to refer children meeting the inclusion criteria to the EFIGRO program. **Figure 1** shows planned flow diagram of the study participants from recruitment to the end of the intervention. Inclusion criteria include primary overweight or obesity status defined according to the International Obesity Task Force (28), aged between 9 and 11 years, and to have at least one parent or caregiver willing to participate in the program sessions. Exclusion criteria include medical conditions or medications that would affect study results or limit physical activity. Moreover, girls

who have already begun to menstruate at baseline are not eligible to participate in the study (**Table 1**).

Table 1. Eligibility criteria for the EFIGRO study.

**Inclusion criteria**
Aged 9-11 years
Overweight or obesity status according to the International Obesity Task Force criteria
To have at least one parent or caregiver willing to participate in the program sessions
**Exclusion criteria**
Not available for assessment session
In girls, begun to menstruate at baseline
Medical conditions or medications that would affect study results or limit physical activity

EFIGRO: the effect of exercise on hepatic fat on overweight children

Before starting the study, a pre-screening appointment will be scheduled with the investigation team to assess eligibility, family needs and commitment. Parents will provide the child's health history and family history, and detailed contact information. After completion of the informed consent process, children will undergo anthropometric screening (height and body mass), and will be selected for inclusion in the study if they are classified as overweight or obese. Moreover, all participants will undergo a complete physical examination by the physician of the study.

Sample size calculation

Hepatic fat fraction is the primary outcome of this study. However, as there was no previous information available in the scientific literature allowing us to perform calculations, the required sample size was determined for secondary outcome variables intimately associated to hepatic fat accumulation, i.e., visceral adiposity, insulin resistance and total body fat. Likewise, we expect that pre-post intervention differences in our design consisting in two experimental groups, control and exercise group, will have a size effect (d-cohen) of 0.5 for visceral fat (N=64 in each group, 80% power and α of 0.05 and 0.7 for insulin resistance and total body fat percent (N=34 in each group, 80% power and α of 0.05). Previous studies have reported losses to follow-up between 4% and 17%. Assuming a maximum loss of follow-up of 20%, we plan to recruit a total

of 160 overweight children, 80 children for each group. These effect size estimates are based on previous studies performed in overweight children with similar age range and a similar intervention program (22, 29, 30).

Randomization

Eligible participants will be randomly assigned after completing the baseline measurements to either the control or exercise groups (Figure 1). Randomization of the participants into control or exercise group will be computer generated using IBM-SPSS-Statistical software. Assessment staff will be blinded to participant randomization assignment.

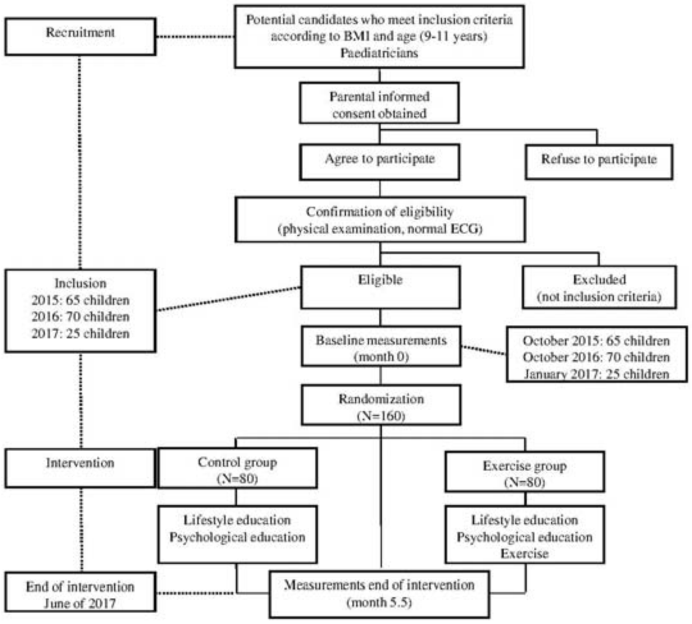

Figure 1.

Exercise training intervention

The exercise group will do exercise 3 days/week, 90 minutes per session, over a 22-week period. The program will be offered to the families from Monday to Friday, five days per week to choose a total of 3 days/week. Sessions will be held in the exercise training facilities of the Faculty of Physical Activity and Sport Sciences of the

University of the Basque Country (Vitoria-Gasteiz, Spain) and will be supervised by exercise specialists. The exercise program consists of cardiovascular endurance and muscle strength exercises. Each session starts with 10 min of warm-up period consisting of two games of 5 min each and will end with 5 min of cool-down period consisting mainly of stretching exercises. The main part of the exercise session consists of 60 minutes of moderate to vigorous aerobic exercises. Individual exercise intensity is calculated from ventilatory thresholds (31) and from the percentage of maximum heart rate (32) obtained from cardiopulmonary exercise test with respiratory gas analyses in treadmill ergometer. The training sessions will be developed to progressively increase the intensity of the aerobic exercises over the 22 weeks. The aerobic workout consists of games and other simple activities and start-and-stop activities. The emphasis of the program will be on intensity and enjoyment. More than 80 activities or games have been selected based on easy of comprehension and fun. These activities include popular running games and some circuits, and have been categorized according to the physical activity intensity. The selection of the activities or games according to their categorization will permit to increase progressively the time on vigorous intensity exercise over the intervention. Finally, each session concludes with 10 minutes of 6-7 resistance exercises in sets of 10-12 repetitions using therabands, fitballs and/or autoloads, involving all major muscle groups (quadriceps, hamstring, abductors, adductors, calves, pectoral, dorsal biceps, triceps and deltoids). Participants will wear a heart rate monitor (Polar RS300X) during the exercise sessions to ensure achievement of the target heart rate zone. Heart rate monitors will be programmed according to individual ventilatory thresholds and % of maximum heart rate.

Rationale and design of the healthy lifestyle education and psychoeducation interventions

Rationale: Obesity is associated with many physical and psychological consequences.

A family-based, lifestyle intervention with a behavioural program aimed at changing dietary habits, physical activity levels and sedentary patterns provide significant decrease of overweight in children as compared to the standard care in the short and in the long-term (16, 33). Moreover, family-based lifestyle interventions can improve psychological well-being in overweight and obese children (19, 34). Therefore, the EFIGRO study will include a family-based healthy lifestyle education program conducted by experienced nutritionists.

Psychological factors are important determinants for behaviour changes in the treatment of obesity in children (33). Moreover, the concept of eating as a means of coping with stressors/negative emotions, i.e., emotional eating, is a mechanism by which emotion regulation relates to obesity (35). Depression, low self-esteem and bullying are much more frequent in overweight and obese children than in their non-overweight peers (17, 36). Therefore, the intervention will include a family-based psychoeducation program leaded and conducted by an experienced psychologist in behaviour changes.

Design: The control and the exercise group will attend both the healthy lifestyle education and psychological education programs. Sessions will be delivered to both parents (or caregivers) and children, separately. Being aware that the intervention program demands time to the families and that this could affect the adherence to the intervention, the healthy lifestyle education and psychoeducation interventions will be developed simultaneously. Likewise, families will come to the facilities (rooms) of the Faculty of Physical Activity and Sport Sciences of the University of the Basque Country 11 times for 90 minutes, once every two weeks, over the intervention. Children will attend first the healthy lifestyle education program (45 minutes/session), while their parents or caregivers are participating in the psychoeducation program (45

minutes/session), and after children will participate in the psychoeducation program, while their parents or caregivers are attending the healthy lifestyle education program.

Aims and content of the healthy lifestyle education program

The family-based healthy lifestyle education program focuses on promoting changes in three lifestyle behaviour areas known as most relevant lifestyle-related risks for obesity and its co-morbidities: diet, physical activity, and stress (37-40). The aim of the program is to increase children's and parents' self-efficacy, knowledge and motivation to adopt healthier dietary habits, increase physical activity level, reduce sedentary behaviour, and reduce stress. To achieve these objectives, the intervention program includes eight sessions combining different aspects of healthy behaviours with the help of teaching materials such as photographs, power point slides, stories, games such as crosswords, riddles, discussions, etc., and three workshops designed by research nutritionists for children and their parents or carers. Key messages will be consistently included across all sessions. The topics included in the program have been selected because of their relationship with hepatic fat deposition or excess adiposity (41-45). Other healthy dietary habits not directly associated with adiposity, but related to cardiovascular disease risk, such as the reduction of the dietary salt content, will be also recommended in the context of a healthy diet, but they do not constitute aims or topics of the program. The content and topics of the sessions are described in **Table 2**.

Table 2. Objectives and topics of the healthy lifestyle education program.

	Topics and aims	Type of session
First session	To learn how to classify foods according to their sugar and fat content	Lesson
Second session	To promote the consumption of a healthy and complete breakfast understanding its importance for health	Lesson
Third session	To promote the increase in daily physical activity level up to 30 min/day and the reduction of time spent on sedentary behaviours such as TV viewing, computer and video games, and smart phones explaining why this is important	Lesson

Fourth session	a) To learn about the importance of eating five times a day for health and body mass control<br>b) To learn about healthy options for morning and afternoon snacks	Lesson
Fifth session	To enhance daily consumption of fruits and vegetables	Workshop:<br>healthy cooking workshop to prepare a common recipe
Sixth session	To reduce the consumption of energy dense foods and sugar sweetened drinks	Workshop:<br>To weigh the sugar content of some foods and beverages typically consumed by the children
Seventh session	To promote the increase in daily physical activity level up to 60 min/day and the reduction of time spent on sedentary behaviours such as TV viewing, computer and video games, and smart phones	Lesson
Eighth session	To distinguish between hunger and appetite<br>to enhance awareness of the importance of an adequate sleep duration	Lesson
Ninth session	To learn and understand the nutritional information of food labelling	Lesson
Tenth session	To clarify certain popular myths related to some foods	Lesson
Eleventh session	To practice and integrate the knowledge acquired	Workshop:<br>to develop complete and healthy menus

Written information emphasizing key messages will be given to parents or caregivers to take home after the workshops. To reinforce the messages delivered in the sessions and workshops and to involve parents or caregivers on lifestyle behaviour changes, there are matching homework activities planned. Likewise, children will have a booklet with 11 chapters, one chapter per lesson, containing three objectives to follow every day. The content and fulfilment of the homework activities will be discussed in the next session.

Aims and content of the psychoeducation intervention

The intervention meetings with parental participation include 11 sessions of 45 minutes aimed to: (a) increase awareness of the problems that could derive from overweight or obese children and (b) provide parental skills to get a favourable family environment in order to make positive changes in their lifestyles and (c) learn assertive communication skills (46). To achieve these objectives, the intervention program consists of sessions

where different topics are presented and discussed, *e.g.*, teasing at school and how to deal with it, expressing emotions and educating their children in managing them or changing habits in the family context.

The intervention program conducted in the participating children also includes 11 sessions of 45 minutes with the following goals: (a) to ease tools to manage the emotions and feelings that they experience because of their condition of being overweight or obese and (b) to provide skills to improve their self-esteem and the psychological and social well-being. In order to reach these aims, the intervention program consists of sessions where different topics are presented and discussed, e.g., teasing at school, putting into words the emotions and feelings that my body makes me feel or activities such as expressing parts of the body that each one likes of herself/himself.

Participant retention and addressing compliance and adherence

New activities on a regular basis will be introduced to maintain interest in the exercise sessions. Children will be invited to suggest appropriate activities. Throughout the project, the investigation team will be aware that success is primarily dependent on enjoyment by the children, because the amount and intensity of the exercise rely on the children's active participation. The staff will continuously try to give the children a positive experience and will celebrate and reward the success achieving the different goals of both the healthy lifestyle education and the exercise programs to motivate the participation and to maintain their interest over the 22 weeks. For instance, working on the target heart rate range in the exercise program or achieving the goal proposed in the healthy lifestyle education program (i.e., having healthy breakfast every day, tasting a different fruit or vegetable every day, etc.) will be rewarded with a smiley emoticon.

The participants will be marked as "absent" if the child does not attend the session or when he/she refuses to participate on proposed games or exercise activities. Parents will

receive telephone calls in case problems pursuing, the child will be dropped from the program to maintain the integrity and the adherence for the other children. The attendance of both children and parents or caregivers (as well as who is attending the session, father, mother, etc.) to the lifestyle education and psychoeducation programs will be also recorded. Regardless of adherence, participants will be encouraged to return to post-testing measurements. The adherence to the exercise, healthy lifestyle education and psychoeducation programs (percentage of attendance) will be used as cofounders in the statistical analyses.

Measurements

All measurements, except potential confounders such as pubertal development and demographic factors, will be conducted at baseline and repeated after 22 weeks of intervention (Figure 1). Post-intervention measurements will be scheduled within three days following the last exercise session or last healthy lifestyle program session in the exercise and in the control group, respectively (**Table 2**).

Table 2. Overview of the measurements and methodology at baseline and post-test in the EFIGRO study

Measure	Methodology
**Primary outcome**	
Hepatic fat (%)	Magnetic resonance imaging
**Secondary outcomes**	
Physical measures	
Pubertal development (Tanner stage)	Physical examination
Body mass (kg)	Scale
Height (cm)	Stadiometer
Waist circumference (cm)	Non-elastic tape
Systolic and diastolic blood pressure (mm Hg)	Oscillometric monitor device
Lean mass (kg)	Dual X-ray absorptiometry
Body fat percentage	Dual X-ray absorptiometry
Bone mineral density (g/cm^2)	Dual X-ray absorptiometry
Abdominal adiposity (g)	Dual X-ray absorptiometry
Truncal adiposity (g)	Dual X-ray absorptiometry
Visceral abdominal adiposity (cm^2)	Magnetic resonance imaging
Subcutaneous abdominal adiposity (cm^2)	Magnetic resonance imaging
Biochemical measures	
Glucose (mg/dL)	Enzymatic spectrophotometry
Insulin, leptin, adiponectine	Enzyme-Linked Immunosorbent Assay
Total-, HDL and LDL-cholesterol (mg/dL)	Enzymatic spectrophotometry
Tryglicerides (mg/dL)	Enzymatic spectrophotometry

Alanine aminotransferase (U/L)	Enzymatic tests
Aspartate aminotransferase (U/L)	Enzymatic tests
Gamma glutamyl transferase (U/L)	Enzymatic tests
C-reactive protein (g/dL)	Enzyme immunoassay
TSH, T3, T4	Radioimmunoassay
Uric acid (mg/dL)	Enzymatic spectrophotometry
Urea, Bilirubine	Enzymatic spectrophotometry
Health-related physical fitness	
Cardiorespiratory fitness	20m shuttle run test<br>Cardiopulmonary exercise test
Muscular fitness	Handgrip strength<br>Standing long jump tests
Speed-agility	4x10m shuttle run test
Sedentary behaviours	Questionnaires
Psychological assessment	Questionnaires
**Potential confounders**	
Pubertal development (Tanner stage)	Physical examination
Dietary assessment	24h recalls and food frequency questionnaires
Physical activity assessment	Accelerometry and questionnaires
Socio-demographic variables	
Socioeconomic status	Questionnaires
Neonatal variables	Health booklets and questionnaires
Family medical history	Questionnaires
Demographic characteristics	Questionnaires

EFIGRO: the effect of exercise on hepatic fat on overweight children; HDL: high density lipoprotein; LDL: low density lipoprotein TSH: thyroid stimulating hormone; T3: triiodothyronine; T4: free tiroxine.

Primary outcome

Hepatic fat

Hepatic fat fraction will be measured by magnetic resonance imaging using a 1.5T system (MAGNETOM Avanto, SiemensHealthcare, Erlangen, Germany) equipped with a phased-array surface coil and a spine array coil and provided by the work-in-progress software package by Siemens Medical System (version syngo.MR B17A) . For hepatic fat quantification, two different 3D gradient-echo sequences will be used in breath-hold: a two-point opposed- and in-phase data acquisition with Dixon water/fat separation, and a prototype six-echo acquisition with advanced signal analysis that provides a more accurate liver fat estimation (47). This algorithm was previously described (47) and validated (48). Liver fat will be quantified as the percentage of relative signal intensity

loss of the liver on opposed-phase images with the two-point Dixon, whereas for the multi-echo acquisition a fat percentage map will be calculated inline using an prototype implementation of a multi-step adaptive fitting approach, taking into account transversal relaxation effects and the spectral complexity of fat (47, 49).

Secondary outcomes measures

The first secondary objective of this study will test the additional effect of aerobic and strength training on cardiometabolic risk factors. Therefore, secondary outcome measures include traditional (total and abdominal adiposity, blood pressure, lipid profile or insulin resistance), a non-traditional cardiometabolic risk factors (visceral adiposity, leptin, adiponectin, liver enzymes, C-reactive protein, uric acid, cardiorespiratory and muscular fitness). Secondary measures also include psychological measurements to evaluate the additional effect of the exercise intervention in self-esteem and well being of overweight and obese children (second secondary objective).

Anthropometry

Body mass is measured to the nearest 0.1 kg (SECA 760) with the children in their underwear. Height will be measured using a stadiometer (SECA 220) to the nearest 5 mm with the children barefoot. Body mass index (BMI) will be calculated as (weight [kg])/(height [m^2]). Waist (narrowest point) will be measured by standard procedures with an anthropometric non-elastic tape (SECA 200) and waist to height ratio is calculated. Anthropometrics will be measured at least twice until consistent measures will be obtained in the Human Exercise Physiology Laboratory at the Faculty of Physical Activity and Sport Sciences of the University of the Basque Country (Vitoria-Gasteiz, Spain).

Body composition

High levels of total, abdominal and visceral adiposity and low levels of lean mass are important cardiovascular disease risk factors. Dual energy X-ray absorptiometry (HOLOGIC, QDR 4500W) of the whole body will be used to estimate fat mass, bone mineral content and density, and bone free lean mass tissue. Abdominal adiposity will be assessed at three different regions using an extended research model as described elsewhere (50).

Visceral and subcutaneous abdominal fat will be measured by magnetic resonance imaging using a 1.5T system (MAGNETOM Avanto, SiemensHealthcare, Erlangen, Germany) equipped with a phased-array surface coil and a spine array coil. Visceral and subcutaneous fat volume will be calculated on the fat-only resultant volumetric images of the six-echo acquisition, from the hepatic dome to the lumbosacral union. An automatic algorithm will segment the subcutaneous and the visceral abdominal fat from the rest of the tissues, particularly bone tissue. This algorithm is based on mathematical morphological operations, watershedding and connected-component labeling. The result of the automatic segmentation will be visually checked and manually corrected when needed.

Blood pressure

Blood pressure measurements will be performed following the recommendations for children (51). Systolic and diastolic blood pressures will be measured by an arm blood pressure oscillometric monitor device (OMRON® M6). Participants will be instructed to be seated with their backs supported and feet on the ground. Two blood pressure readings will be taken with a 10-min interval in-between and the lowest reading will be recorded.

Biochemical variables

Morning fasting blood samples will be obtained from each child by experienced nursing staff in the Clinical Trials Unit of TECNALIA (HUA, Vitoria-Gasteiz). Blood samples will be immediately centrifuged, aliquoted within one hour collection and stored at -70 ºC or below. This permits measurements of glucose, insulin, lipid profile (total-, HDL- and LDL-cholesterol, and TG), liver enzymes (ALT, AST and gamma-GT), leptin, adiponectin, C-reactive protein, thyroid hormones (TSH, T3 and T4), urea, bilirubin and uric acid.

Physical fitness

Physical fitness includes cardiorespiratory fitness, muscular strength and speed agility. A high level of physical fitness is associated with cardiovascular health in children (52). Moreover, changes in cardiorespiratory fitness in response to exercise could be good predictors of changes in cardiovascular disease risk factors (27).

Physical fitness will be assessed using valid and reliable tests for young people (53, 54) (more information on www.thealphaproject.net). All the tests will be performed twice and the best score will be recorded except cardiorespiratory tests, which will be performed only once. Muscular fitness will be assessed by means of the handgrip strength and standing long jump tests (55). Speed-agility will be assessed with the 4x10m shuttle run test (56). Cardiorespiratory fitness will be assessed by two different tests: (a) The 20m shuttle run test (57) in which the equation reported by Léger et al. (57) will be used to estimate the maximum oxygen consumption (VO_{2max}, ml/kg/min) from the 20m shuttle run test scores, and (b) Direct cardiopulmonary exercise progressive incremental treadmill test using the modified American College of Sports Medicine protocol with respiratory gas analysis to exhaustion (32). The test starts with a 6% slope and increases by 1% per minute with a fixed speed of 4.8 km/h (3.0 mph) (Treadmill Ergelek, Vitoria-Gasteiz, Spain). Children will be familiarized with the

treadmill ergometer prior to testing. Children will be encouraged to walk to the limit of their tolerance during the test. Metabolic gas exchange will be measured breath by breath throughout the test using a metabolic cart (monitor Ergo CardMedi-soft S.S, Belgium Ref. USM001 V1.0), and data will be averaged each 60 seconds. Heart rate will be monitored at rest, continuously throughout the test, and for 5 minutes in the recovery period using a 3-lead electrocardiogram (Med Card Stress MEDISOFT). The exercise electrocardiogram will be monitored continuously. Blood pressure will be measured at rest and three times in the recovery period (minutes 1, 3 and 5). Children will report their perceived exertion according to the self-reported rating (from 0 to 10) of perceived exertion Omni-scale (58) at the end of each minute. True maximum oxygen consumption (VO_{2max}) will be verified using the American College of Sports Medicine exercise testing criteria. The exercise test will continue until the child decides to stop or until VO_{2max} is reached. Participants who do not achieve these criteria will be classified as having reached the VO_{2peak}. Subsequent analysis will account for this factor. The exercise test will be supervised by a physician and the result of the electrocardiogram obtained during the test reviewed by a cardiologist to ensure that there are no medical conditions that limit or advise against the participation in the exercise program.

The cardiopulmonary exercise test will be used to obtain objective and direct measurements of peak aerobic capacity and peak heart rate, and to determine ventilatory thresholds. Exercise intensities are established according to two different criteria: individual ventilatory thresholds (first ventilatroy threshold, VT1, and second ventilator threshold, VT2) and percentages of HR_{max}. The VT1 can be determined by analyzing the slope of the carbon dioxide production (VCO_2) *vs.* VO_2 relationship, identifying as the point of transition in the VCO_2 *vs.* VO_2 slope from less than 1 to more than 1, or by

the ventilator equivalent of VO_2 *vs.* work rate relationship, identifiable as the nadir of the relationship. The VT2 is identifiable as the nadir of the ventilator equivalent ratio of VCO_2 *vs.* work rate relationship. The identification of the two ventilatory thresholds will determine the different exercise intensity domains: (a) light to moderate exercise intensity when HR values are below VT1; (b) moderate to high or vigorous exercise intensity if HR values are between VT1 and VT2, and (c) high to severe intensity exercise intensity when HR values are from VT2 to peak intensity. When the VT2 is not possible to identify, exercise intensity domains will be established taking into account the percentages of HR_{max}, *i.e.,* moderate intensity is defined between 64% and 75 % of HR_{max}, vigorous intensity from ≥76% to <96 % of HR_{max} and near to maximal from ≥96% to 100% HR_{max} (32).

Exercise intensities are established according to the percentages of maximum heart rate as follows: moderate intensity is defined between 64% and 75 % of maximum heart rate, vigorous intensity from ≥76% to <96 % of maximum heart rate, and near to maximal from ≥96% to 100% maximum heart rate (32).

Psychological measures

Changes in the psychological variables will be assessed to examine the additional effect of aerobic and strength training on self-esteem and psychological well-being in overweight and obese children.

The psychological variables will be estimated with valid and reliable questionnaires and include: anxiety, daily stress, self-concept, depression and the perception of parental feeding styles in children and parenting practices and eating behaviour in parents.

The questionnaires for children include the Spanish version of the State-Trait Anxiety Inventory for Children (59), the Children’s Daily Stressors Inventory (60), the Self-concept form-5 Questionnaire (61), the translated version of the Children’s Depression

Inventory (62) and the adapted Spanish version of Parental Feeding Styles Questionnaire (63). The State-Trait Anxiety Inventory for Children measures chronic symptoms of anxiety and contains 20 items asking the frequency in which children experience anxiety symptoms such as “I am scared”, “I feel troubled” and “I get a funny feeling in my stomach” using three-point scales: 1= *almost never*, 2= *sometimes* and 3= *often*. A total trait anxiety score is calculated by summing the ratings for all items. The Children’s Daily Stressors Inventory assesses the daily stress referring to the worries and setbacks that occur in the everyday lives of school-age children. This self-report inventory includes 22 dichotomous items covering the areas of health and psychosomatic problems, stress at school and stress in family context. The score is obtained by adding the total of positive answers. Higher scores indicate children experienced more stress. The Self-concept form-5 Questionnaire is a questionnaire for measuring the self-concept in five dimensions: (a) Academic self-concept refers to the personal beliefs about the quality of the performance as a student, (b) Social self-concept is the perception of the performance in social relations, (c) Emotional self-concept is one’s understanding of his/her emotional state as well as his/her responses to specific situations when certain degree of commitment and involvement is necessary in everyday life, (d) Family self-concept refers to the children’s perception in his/her involvement, participation and integration in the family environment, and (e) Physical self-concept is one’s beliefs about his/her physical appearance and his/her physical condition. The Self-concept form-5 Questionnaire contains 30 statements on a scale from 0 *(Totally disagree)* to 99 *(Totally agree)*. The Children’s Depression Inventory assesses the severity of self-reported symptoms of childhood depression. Subscales include negative mood, interpersonal difficulties, ineffectiveness, negative self-esteem, and anhedonia. These items can be assessed on a 3-point scale: 0 (*absence of*

symptomatology), 1 (*mild symptomatology*), or 2 (*severe symptomatology*). The total score ranges between 0 and 54 points. Finally, the Parental Feeding Styles Questionnaire is used to measure children's perception of the feeding styles shown by parents. This questionnaire contains 26 items and includes four subscales (i.e., Emotional feeding, Instrumental feeding, Control, and Encouragement). The Parental Feeding Styles Questionnaire can be answered on a five-point Likert scale ranging from 1 *(Never)* to 5 *(Always)* according to the frequency in which the situation described happened. The higher the score, the higher the feeding style at issue.

The questionnaires for parents include the Parent-Child Relationship Inventory (64) and the Three Factor Eating Questionnaire(65). The Parent-Child Relationship Inventory assesses parents' attitudes toward parenting and toward their children. The Parent-Child Relationship Inventory is a 78-items self-report questionnaire answered in a four point Likert scale ranging from 1 *(Strongly agree)* to 4 *(Totally disagree).* The statements considered in this questionnaire are grouped into seven scales: (a) Social and emotional support, (b) Satisfaction with parenting, (c) Involvement, (d) Communication, (e) Limit setting, (f) Autonomy, and (g) Role orientation. High scores on the different scales indicate greater agreement with the situation defined in each scale. The Three Factor Eating Questionnaire is a 51-item self-report questionnaire that assesses three different dimensions of eating behaviour: (a) Cognitive Restraint, (b) Disinhibition, and (c) Hunger. The questionnaire has two parts; in the first part, participants answer 36 statements with *true* or *false*. In the second part, participants are asked to circle the response appropriate to them. Scores can range from 0 to 21 for the restraint scale, from 0 to 16 for the disinhibition scale and from 0 to 14 for the hunger scale.

Potential confounders

Pubertal development

Pubertal development will be directly assessed according to Tanner criteria (genital development and pubic hair) by the paediatricians in the Paediatric Endocrinology Unit of the HUA.

Physical activity, sedentary behaviour and dietary assessment

Both the control and the exercise groups will attend the same healthy lifestyle education program. However, differences in changes in physical activity level, sedentary behaviour and dietary habits could be potential confounders on the effect of the intervention on hepatic fat and cardiometabolic risk factors.

Physical activity will be objectively measured by accelerometry. Children will be asked to wear an accelerometer on their non-dominant wrist (wActisleep-BT, USA) for seven consecutive days all the time, except for water-based activities. Thresholds will be used to classify raw counts per minute into intensities of physical activity (66). Information about sleep duration and quality will be also obtained from the accelerometers.

Participants will also complete a diary log. A self-reported sedentary behaviour questionnaire (67) will be administered to the children. Participants will report the frequency of specified sedentary behaviours using predefined response categories separately for weekdays and weekends. Behaviours to be assessed include watching TV, playing computer games, playing video games or phone games, surfing the Internet for recreation, surfing the Internet for study purposes, and studying (nonschool time).

Dietary intake will be evaluated by two non-consecutive 24h recalls within a period of seven days. Children will report all their food and beverage ingestions assisted by nutritionists. Moreover, dietary habits and patterns will be evaluated by two different food frequency questionnaires: the KidMed index for the children (68) and a validated food frequency questionnaire for the parents or caregivers (69). The KidMed index was

developed to estimate adherence to the Mediterranean dietary pattern in children and young adults, based on foods/dietary patterns that sustain it.

Socio-demographic variables

A parental questionnaire will be used to collect information about socioeconomic status, demographic characteristics, neonatal data of the children and family medical history of obesity, diabetes, dyslipidemia and hypertension. The Family Affluence Scale will be used as an index of socioeconomic status. The Family Affluence Scale is an indicator of affluence *versus* material deprivation and describes family expenditure and consumption based on 4 items: having an own bedroom, number of cars per family, number of PCs at home and internet access(70). The Family Affluence Scale has been widely used as an index of socioeconomic status(71, 72). Other indicators of socioeconomic status will be used: the parental education level (primary school, secondary school/technical training or university degree), and parental occupation according to the International Standard Classification of Occupation (http://www.ilo.org/public/english/bureau/stat/isco/index.htm). Demographic characteristics will include date of birth, sex, ethnicity and family structure. Perinatal data will include weight (g), length (cm) and head circumference at birth (cm), gestational age (weeks), and duration of both breastfeeding and exclusive breastfeeding (months).

Assessment of side effects

We will record adverse effects or health problems attributable to the testing sessions or exercise sessions. Particularly we will report those attributable to the exercise sessions such as muscle pain, fatigue, and general aches and pains by self-report during the study period and the occurrence of anxiety, depression or disordered eating or behaviors by self-report or parental report. An independent researcher will be in charge of auditing all assessment staff to record all these events in the participants over the study period.

Data analysis plan

The data analysis plan will be defined based mainly in two analyses: i) the per-protocol analysis, and ii) the intention-to-treat analysis (ITT). For reporting the main findings, we will use the per-protocol principle which includes children with overweight or obesity that completed pre and post intervention assessments, and attend at least 50% of the family-based lifestyle program (no minimum required for exercise program). We will focus on per-protocol principle because we are interested in knowing the “real” efficacy rather than effectiveness of our intervention. In addition to the per-protocol principle, we will also analyse the data using the ITT. Under this principle, we will use multiple imputation for observations lost at post intervention.

The baseline characteristics of the subjects’ in the two intervention groups will be compared using either the Student t test (for continuous variables) or the x^2 test (for categorical variables). Differences between the intervention groups in terms of postintervention values for the primary and secondary outcomes will be also examined using the Student t test. Within group differences (pre vs. post values) in primary and secondary outcomes will be examined using the Student paired t test following per protocol and intention-to treat principles. In both the per protocol and the intention-to-treat analyses, differences between the control and exercise groups in terms of changes in primary and secondary outcomes will be examined by ANCOVA adjusting for baseline values. Cohen’s d will be used to estimate the effect size and 95% CI.

Significance will be set at $P < 0.05$. All calculations will be made using the Statistical Package for Social Sciences version 24.0 for Windows (SPSS Inc. Chicago, IL). We will also use R software (v.3.1.2) for analysis and figures.

Discussion

The worldwide dramatic rise in obesity prevalence, along with its metabolic consequences, makes NAFLD a potential leading cause of liver disease, diabetes, cardiovascular disease and increased risk of all-cause of mortality (73). Weight loss is the single most beneficial treatment for overweight adults with NAFLD (74). Several studies reported that body mass loss of 5%-10% improves liver steatosis and inflammation (75). Lifestyle interventions focused on weight loss through a combination of energy restricted diet and exercise can be difficult to implement, particularly in the long term, but, when achieved, they are effective. In children, weight loss is very difficult to achieve in practice, even in the short term. Moreover, in prepubescent children the objective in the treatment of excess adiposity is body mass maintenance, rather than weight loss, allowing an improvement of BMI during growth. However, cardiovascular disease risk factors such as insulin resistance, elevated blood pressure, high levels of cholesterol and TG and NAFLD are present in obese children and should be treated (4, 76). As an alternative to dietary restriction to promote body mass loss, previous investigations have shown that exercise is able to reduce hepatic fat with minimal or no weight loss in adolescents (25-27) and to improve cardiovascular disease risk factors without caloric restriction in children (22, 30). Likewise, the inclusion of a supervised exercise program in a multidisciplinary intervention seems to be a good alternative to body mass loss in the treatment and prevention of obesity related comorbidities in children.

Currently, no specific clinical recommendation and lifestyle interventions guidelines are available for the prevention and management of NAFLD (77). The results of this trial will provide evidence regarding the effectiveness of a multidisciplinary, family involved, intervention including exercise in the reduction of hepatic steatosis and

cardiovascular disease risk factors without caloric restriction in overweight children, which could be useful in clinical practice.

ACKNOWLEDGMENTS

Our special thanks go to the families, children and parents, who are participating in the study for their collaboration. We also acknowledge the paediatricians for their participation in the recruitment, the members involved in the assessments for their efforts and to Siemens Medical Systems for supplying the software to quantify hepatic fat, and to the pre- and post-graduate students for their collaboration in the exercise training sessions. We would like also to acknowledge the collaboration of Antonio de Blas, head of the "Comarca de Araba, Osakidetza" and the team of school health.

Competing Interests

The authors declare that they have no competing interest.

REFERENCES

1. Wijnhoven TM, van Raaij JM, Spinelli A, Rito AI, Hovengen R, Kunesova M, et al. WHO European Childhood Obesity Surveillance Initiative 2008: weight, height and body mass index in 6-9-year-old children. Pediatr Obes. 2013;8(2):79-97.

2. Ortega FB, Ruiz JR, Labayen I, Martinez-Gomez D, Vicente-Rodriguez G, Cuenca-Garcia M, et al. Health Inequalities in Urban Adolescents: Role of Physical Activity, Diet, and Genetics. Pediatrics. 2014;133(4):E884-E95.

3. Weiss R, Bremer AA, Lustig RH. What is metabolic syndrome, and why are children getting it? Ann N Y Acad Sci. 2013;1281:123-40.

4. Welsh JA, Karpen S, Vos MB. Increasing prevalence of nonalcoholic fatty liver disease among United States adolescents, 1988-1994 to 2007-2010. J Pediatr. 2013;162(3):496-500.e1.

5. Schwimmer JB, Pardee PE, Lavine JE, Blumkin AK, Cook S. Cardiovascular risk factors and the metabolic syndrome in pediatric nonalcoholic fatty liver disease. Circulation. 2008;118(3):277-83.

6. Schwimmer JB, McGreal N, Deutsch R, Finegold MJ, Lavine JE. Influence of gender, race, and ethnicity on suspected fatty liver in obese adolescents. Pediatrics. 2005;115(5):e561-5.

7. Mencin AA, Lavine JE. Nonalcoholic fatty liver disease in children. Curr Opin Clin Nutr Metab Care. 2011;14(2):151-7.

8. Schwimmer JB, Deutsch R, Kahen T, Lavine JE, Stanley C, Behling C. Prevalence of fatty liver in children and adolescents. Pediatrics. 2006;118(4):1388-93.

9. Schwimmer JB, Dunn W, Norman GJ, Pardee PE, Middleton MS, Kerkar N, et al. SAFETY study: alanine aminotransferase cutoff values are set too high for reliable detection of pediatric chronic liver disease. Gastroenterology. 2010;138(4):1357-64, 64 e1-2.

10. Berardis S, Sokal E. Pediatric non-alcoholic fatty liver disease: an increasing public health issue. Eur J Pediatr. 2014;173(2):131-9.

11. Schwimmer JB, Zepeda A, Newton KP, Xanthakos SA, Behling C, Hallinan EK, et al. Longitudinal assessment of high blood pressure in children with nonalcoholic fatty liver disease. PLoS One. 2014;9(11):e112569.

12. Al Rifai M, Silverman MG, Nasir K, Budoff MJ, Blankstein R, Szklo M, et al. The association of nonalcoholic fatty liver disease, obesity, and metabolic syndrome, with systemic inflammation and subclinical atherosclerosis: The Multi-Ethnic Study of Atherosclerosis (MESA). Atherosclerosis. 2015.

13. Labayen I, Ruiz JR, Ortega FB, Davis CL, Rodriguez G, Gonzalez-Gross M, et al. Liver enzymes and clustering cardiometabolic risk factors in European adolescents: the HELENA study. Pediatr Obes. 2015; doi: 10.1111/ijpo.273.

14. Armstrong MJ, Newsome PN. Editorial: treatment for NASH - helping the liver or helping the heart? Aliment Pharmacol Ther. 2015;41(5):487.

15. Turner KM, Salisbury C, Shield JP. Parents' views and experiences of childhood obesity management in primary care: a qualitative study. Fam Pract. 2012;29(4):476-81.

16. Spear BA, Barlow SE, Ervin C, Ludwig DS, Saelens BE, Schetzina KE, et al. Recommendations for treatment of child and adolescent overweight and obesity. Pediatrics. 2007;120 Suppl 4:S254-88.

17. De Niet JE, Naiman DI. Psychosocial aspects of childhood obesity. Minerva Pediatr. 2011;63(6):491-505.

18. van der Kruk JJ, Kortekaas F, Lucas C, Jager-Wittenaar H. Obesity: a systematic review on parental involvement in long-term European childhood weight control interventions with a nutritional focus. Obes Rev. 2013;14(9):745-60.

19. Daley AJ, Copeland RJ, Wright NP, Roalfe A, Wales JK. Exercise therapy as a treatment for psychopathologic conditions in obese and morbidly obese adolescents: a randomized, controlled trial. Pediatrics. 2006;118(5):2126-34.

20. Marcason W. What are the current guidelines for pediatric non-alcoholic fatty liver disease? J Acad Nutr Diet. 2013;113(12):1772.

21. Dixon JB, Bhathal PS, Hughes NR, O'Brien PE. Nonalcoholic fatty liver disease: Improvement in liver histological analysis with weight loss. Hepatology. 2004;39(6):1647-54.

22. Davis CL, Pollock NK, Waller JL, Allison JD, Dennis BA, Bassali R, et al. Exercise dose and diabetes risk in overweight and obese children: a randomized controlled trial. JAMA. 2012;308:1103-12.

23. Escalante Y, Saavedra JM, Garcia-Hermoso A, Dominguez AM. Improvement of the lipid profile with exercise in obese children: a systematic review. Prev Med. 2012;54(5):293-301.

24. Garcia-Hermoso A, Saavedra JM, Escalante Y. Effects of exercise on resting blood pressure in obese children: a meta-analysis of randomized controlled trials. Obes Rev. 2013;14(11):919-28.

25. Lee S, Bacha F, Hannon T, Kuk JL, Boesch C, Arslanian S. Effects of aerobic versus resistance exercise without caloric restriction on abdominal fat, intrahepatic lipid, and insulin sensitivity in obese adolescent boys: a randomized, controlled trial. Diabetes. 2012;61(11):2787-95.

26. van der Heijden GJ, Wang ZJ, Chu ZD, Sauer PJ, Haymond MW, Rodriguez LM, et al. A 12-week aerobic exercise program reduces hepatic fat accumulation and insulin resistance in obese, Hispanic adolescents. Obesity (Silver Spring). 2010;18(2):384-90.

27. Senechal M, Rempel M, Duhamel TA, MacIntosh AC, Hay J, Wicklow B, et al. Fitness is a determinant of the metabolic response to endurance training in adolescents at risk of type 2 diabetes mellitus. Obesity (Silver Spring). 2015;23(4):823-32.

28. Cole TJ, Lobstein T. Extended international (IOTF) body mass index cut-offs for thinness, overweight and obesity. Pediatr Obes. 2012;7(4):284-94.

29. Ferguson MA, Gutin B, Le NA, Karp W, Litaker M, Humphries M, et al. Effects of exercise training and its cessation on components of the insulin resistance syndrome in obese children. Int J Obes Relat Metab Disord. 1999;23(8):889-95.

30. Owens S, Gutin B, Allison J, Riggs S, Ferguson M, Litaker M, et al. Effect of physical training on total and visceral fat in obese children. Med Sci Sports Exerc. 1999;31(1):143-8.

31. Quinart S, Mourot L, Negre V, Simon-Rigaud ML, Nicolet-Guenat M, Bertrand AM, et al. Ventilatory thresholds determined from HRV: comparison of 2 methods in obese adolescents. Int J Sports Med. 2014;35(3):203-8.

32. Thompson PD, Arena R, Riebe D, Pescatello LS. ACSM's new preparticipation health screening recommendations from ACSM's guidelines for exercise testing and prescription, ninth edition. Curr Sports Med Rep. 2013;12:215-7.

33. Oude Luttikhuis H, Baur L, Jansen H, Shrewsbury VA, O'Malley C, Stolk RP, et al. Interventions for treating obesity in children. Cochrane Database Syst Rev. 2009(1):Cd001872.

34. Eime RM, Young JA, Harvey JT, Charity MJ, Payne WR. A systematic review of the psychological and social benefits of participation in sport for children and adolescents: informing development of a conceptual model of health through sport. Int J Behav Nutr Phys Act. 2013;10:98.

35. Graziano PA, Calkins SD, Keane SP. Toddler self-regulation skills predict risk for pediatric obesity. Int J Obes (Lond). 2010;34(4):633-41.

36. Castillo F, Francis L, Wylie-Rosett J, Isasi CR. Depressive symptoms are associated with excess weight and unhealthier lifestyle behaviors in urban adolescents. Child Obes. 2014;10(5):400-7.

37. Thompson F, Subar A. Dietary Assessment Methodology. In: Coulston A, Boushey C, editors. Nutrition in the Prevention and Treatment of Disease. 2nd ed. San Diego: Elsevier Academic Press; 2008. p. 3-39.

38. Lobstein T, Jackson-Leach R, Moodie ML, Hall KD, Gortmaker SL, Swinburn BA, et al. Child and adolescent obesity: part of a bigger picture. Lancet. 2015;20;385(9986):2510-20.

39. Hill JO, Wyatt HR, Reed GW, Peters JC. Obesity and the environment: where do we go from here? Science. 2003;299(5608):853-5.

40. Kunin-Batson AS, Seburg EM, Crain AL, Jaka MM, Langer SL, Levy RL, et al. Household factors, family behavior patterns, and adherence to dietary and physical activity guidelines among children at risk for obesity. J Nutr Educ Behav. 2015;47(3):206-15 e1.

41. Hallstrom L, Labayen I, Ruiz JR, Patterson E, Vereecken CA, Breidenassel C, et al. Breakfast consumption and CVD risk factors in European adolescents: the HELENA (Healthy Lifestyle in Europe by Nutrition in Adolescence) Study. Public Health Nutr. 2013;16(7):1296-305.

42. Cook LT, O'Reilly GA, Goran MI, Weigensberg MJ, Spruijt-Metz D, Davis JN. Vegetable consumption is linked to decreased visceral and liver fat and improved insulin resistance in overweight Latino youth. J Acad Nutr Diet. 2014;114(11):1776-83.

43. Bornhorst C, Wijnhoven TM, Kunesova M, Yngve A, Rito AI, Lissner L, et al. WHO European Childhood Obesity Surveillance Initiative: associations between sleep duration, screen time and food consumption frequencies. BMC Public Health. 152015. p. 442.

44. Franckle RL, Falbe J, Gortmaker S, Ganter C, Taveras EM, Land T, et al. Insufficient sleep among elementary and middle school students is linked with elevated soda consumption and other unhealthy dietary behaviors. Prev Med. 2015;74:36-41.

45. Mollard RC, Senechal M, MacIntosh AC, Hay J, Wicklow BA, Wittmeier KD, et al. Dietary determinants of hepatic steatosis and visceral adiposity in overweight and obese youth at risk of type 2 diabetes. Am J Clin Nutr. 2014;99:804-12.

46. Hemmingsson E. A new model of the role of psychological and emotional distress in promoting obesity: conceptual review with implications for treatment and prevention. Obes Rev. 2014;15(9):769-79.

47. Zhong X, Nickel MD, Kannengiesser SA, Dale BM, Kiefer B, Bashir MR. Liver fat quantification using a multi-step adaptive fitting approach with multi-echo GRE imaging. Magn Reson Med. 2014;72(5):1353-65.

48. Bashir MR, Zhong X, Nickel MD, Fananapazir G, Kannengiesser SA, Kiefer B, et al. Quantification of hepatic steatosis with a multistep adaptive fitting MRI approach: prospective validation against MR spectroscopy. AJR Am J Roentgenol. 2015;204(2):297-306.

49. Kukuk GM, Hittatiya K, Sprinkart AM, Eggers H, Gieseke J, Block W, et al. Comparison between modified Dixon MRI techniques, MR spectroscopic relaxometry, and different histologic quantification methods in the assessment of hepatic steatosis. Eur Radiol. 2015.

50. Labayen I, Ruiz JR, Vicente-Rodriguez G, Turck D, Rodriguez G, Meirhaeghe A, et al. Early Life programming of Abdominal Adiposity in Adolescents: The HELENA Study. Diabetes Care. 2009;32(11):2120-2.

51. NHBPEP. National High Blood Pressure Education Program Working Group on High Blood Pressure in Children and Adolescents. The fourth report on the diagnosis, evaluation, and treatment of high blood pressure in children and adolescents. Pediatrics. 2004;114(2 Suppl 4th Report):555-76.

52. Ruiz JR, Huybrechts I, Cuenca-Garcia M, Artero EG, Labayen I, Meirhaeghe A, et al. Cardiorespiratory fitness and ideal cardiovascular health in European adolescents. Heart. 2015;101: 766-73.

53. Ruiz JR, Castro-Pinero J, Espana-Romero V, Artero EG, Ortega FB, Cuenca MM, et al. Field-based fitness assessment in young people: the ALPHA health-related fitness test battery for children and adolescents. Br J Sports Med. 2011;45(6):518-24.

54. Castro-Pinero J, Artero EG, Espana-Romero V, Ortega FB, Sjostrom M, Suni J, et al. Criterion-related validity of field-based fitness tests in youth: a systematic review. Br J Sports Med. 2010;44(13):934-43.

55. Ruiz JR, Espana-Romero V, Ortega FB, Sjöström M, Castillo MJ, Gutiérrez A. Hand span influences optimal grip span in male and female teenagers. J Hand Surg [Am]. 2006;31(8):1367-72.

56. Vicente-Rodriguez G, Rey-Lopez JP, Ruiz JR, Jimenez-Pavon D, Bergman P, Ciarapica D, et al. Interrater reliability and time measurement validity of speed-agility field tests in adolescents. J Strength Cond Res. 2011;25(7):2059-63.

57. Leger LA, Mercier D, Gadoury C, Lambert J. The multistage 20 metre shuttle run test for aerobic fitness. J Sports Sci. 1988;6(2):93-101.

58. Suminski RR, Robertson RJ, Goss FL, Olvera N. Validation of the Omni Scale of Perceived Exertion in a sample of Spanish-speaking youth from the USA. Percept Mot Skills. 2008;107(1):181-8.

59. Spielberger CD, Edwards CD. STAIC: Cuestionario de autoevaluación ansiedad estado/rasgo en niños: Manual. Madrid: TEA Ediciones; 1990.

60. Trianes MV, Blanca MJ, Fernández FJ, Escobar M, Maldonado EF, Muñoz AM. Evaluación del estrés infantil: Inventario Infantil de Estresores Cotidianos (IIEC). Psicothema. 2009;21(4):598-603.

61. García F, Musitu G. AF5: Autoconcepto forma 5. Madrid: TEA Ediciones; 2001.

62. Kovacs M, del Barrio V, Ortiz MAC. CDI: Inventario de depresión infantil: Manual: TEA; 2004.

63. Wardle J, Sanderson S, Guthrie CA, Rapoport L, Plomin R. Parental feeding style and the inter-generational transmission of obesity risk. Obesity. 2002;10(6):453-62.

64. Gerard AB. Parent-child relationships inventory (PCRI) manual. Los Angeles: WPS; 1994.

65. Stunkard AJ, Messick S. The three-factor eating questionnaire to measure dietary restraint, disinhibition and hunger. Journal of psychosomatic research. 1985;29(1):71-83.

66. Chandler JL, Brazendale K, Beets MW, Mealing BA. Classification of physical activity intensities using a wrist-worn accelerometer in 8-12-year-old children. Pediatr Obes. 2015. doi: 10.1111/ijpo.12033

67. Rey-Lopez JP, Vicente-Rodriguez G, Ortega FB, Ruiz JR, Martinez-Gomez D, De Henauw S, et al. Sedentary patterns and media availability in European adolescents: The HELENA study. Prev Med. 2010;51(1):50-5.

68. Serra-Majem L, Ribas L, Ngo J, Ortega RM, Garcia A, Perez-Rodrigo C, et al. Food, youth and the Mediterranean diet in Spain. Development of KIDMED, Mediterranean Diet Quality Index in children and adolescents. Public Health Nutr. 2004;7(7):931-5.

69. Vereecken CA, Maes L. A Belgian study on the reliability and relative validity of the Health Behaviour in School-Aged Children food-frequency questionnaire. Public Health Nutr. 2003;6(6):581-8.

70. Currie CE, Elton RA, Todd J, Platt S. Indicators of socioeconomic status for adolescents: the WHO Health Behaviour in School-aged Children Survey. Health Educ Res. 1997;12(3):385-97.

71. Currie C, Molcho M, Boyce W, Holstein B, Torsheim T, Richter M. Researching health inequalities in adolescents: the development of the Health Behaviour in School-Aged Children (HBSC) family affluence scale. Soc Sci Med. 2008;66(6):1429-36.

72. Jimenez Pavon D, Ortega FP, Ruiz JR, Espana Romero V, Garcia Artero E, Moliner Urdiales D, et al. Socioeconomic status influences physical fitness in European adolescents independently of body fat and physical activity: the HELENA study. Nutr Hosp. 2010;25(2):311-6.

73. Than NN, Newsome PN. A concise review of non-alcoholic fatty liver disease. Atherosclerosis. 2015;239(1):192-202.

74. Promrat K, Kleiner DE, Niemeier HM, Jackvony E, Kearns M, Wands JR, et al. Randomized controlled trial testing the effects of weight loss on nonalcoholic steatohepatitis. Hepatology. 2010;51(1):121-9.

75. Vilar-Gomez E, Martinez-Perez Y, Calzadilla-Bertot L, Torres-Gonzalez A, Gra-Oramas B, Gonzalez-Fabian L, et al. Weight Loss via Lifestyle Modification

Significantly Reduces Features of Nonalcoholic Steatohepatitis. Gastroenterology. 2015;149(2):367-378.

76. Schwimmer JB, Deutsch R, Rauch JB, Behling C, Newbury R, Lavine JE. Obesity, insulin resistance, and other clinicopathological correlates of pediatric nonalcoholic fatty liver disease. J Pediatr. 2003;143(4):500-5.

77. AlKhater SA. Paediatric non-alcoholic fatty liver disease: an overview. Obes Rev. 2015;16(5):393-405.

PARTICIPANTS INFORMATIVE SHEET AND INFORMED CONSENT

"EFIGRO" RESEARCH PROJECT:

"Effects of a physical exercise program on liver fat in overweight boys and girls"

PRINCIPAL INVESTIGATOR: Idoia Labayen

INFORMATION TO PARENT / GUARDIAN OF PARTICIPANT

Dear friend:

This is a research project in which we try to examine whether a 6-month exercise program is capable of reducing cardiovascular risk, specifically liver-localized fat, in overweight children. We collaborate with doctors and researchers from the Hospitals of Txagorritxu and Santiago Apóstol, OSATEK, University of the Basque Country and LEIA-TECNALIA.

Overweight and obesity in childhood are associated with an increased risk of some diseases in adulthood, such as cardiovascular disease and type 2 diabetes mellitus. However, the origin of these complications is found in the early stages of life and some of the injuries that cause cardiovascular disease are present as early as childhood. Among the most serious complications are hepatic steatosis (fatty liver) and insulin resistance. It appears that slightly more than 1/3 of overweight children have hepatic steatosis.

Nowadays, there is enough scientific evidence to affirm that the practice of physical exercise has benefits on health and longevity. The results of different studies show that physical exercise programs are capable of reducing fat mass and CV risk factors in children.

WHAT ARE THE OBJECTIVES OF THE STUDY?

Our objective in this work is to examine whether through physical exercise we can reduce liver fat, and also increase insulin sensitivity and improve body composition by increasing the amount of muscle mass.

For this we have proposed a physical exercise program that will be directed by specialists in physical exercise (graduates / graduates in Physical Activity and Sports Sciences) and an education program in healthy lifestyles that will be carried out by qualified Nutritionists.

If you want your child to participate in this research completely voluntarily and completely free of charge, you must be informed about the tests that will be carried out at the beginning and end of the program, as well as the design of the exercise program.

WHAT DOES THE STUDY CONSIST OF?

The physical exercise program:

The physical exercise program will last 60 minutes, 4 days a week for 6 months. The first 5 minutes will be dedicated to the explanation of the activity (description of the exercise, rules or rules of the game, etc.) and the placement of the heart rate monitors to record the heart rate, so the total effective duration of the exercise is 55 minutes. Boys and girls will wear a heart rate monitor during the sessions. The first 5 min are dedicated to warming up through various sports activities and the last 5 min to recovery. After warming up, the first 35 min are dedicated to moderate to vigorous aerobic activities in which the intensity will be progressively increased. The last 10 min include exercises for muscle strength and flexibility that will be performed with appropriate material. The design of the proposed program follows the international recommendations for physical activity for this age group.

The educational program consists of 2 sessions per month of about 30 minutes in length in which the boys and girls and their parents or guardians will receive information about healthy habits and will also be given emotional support.

Methodology

The design of the program is as follows: half of the boys and girls will start the physical exercise and lifestyle education program simultaneously (group 1). The other half of the children will be assigned to a control group that will receive the educational program on healthy lifestyles and will begin with the educational program (group 2, control). Once the 6-month period is over, the control group will be offered participation in the exercise program with the same duration (6 months) and frequency (4 days / week, 60 minutes a day). The allocation of the groups is random.

It is not foreseen to remunerate the participation, although we have prepared some gifts that help us to stimulate their participation and maintenance within the program such as t-shirts and backpacks.

Tests to be carried out

The following tests will be performed on 2 occasions, at the beginning and at the end of the 6-month period.

1) Complete medical review (examination, medical history and blood extraction)

2) Measurement of body composition by dual X-ray absorptiometry (see attached sheet).

3) Evaluation of aerobic capacity: exercise test by bicycle. Children will get on a bike, be monitored, and start pedaling. Slowly your speed and stamina will increase. They will be monitored at all times by electrocardiogram and oxygen consumption will be measured. During the test, a doctor will be present.

4) Measurement of visceral and liver fat: this test is performed using magnetic resonance imaging (MRI). MRI is a non-invasive method without ionizing radiation. MRI is the only imaging modality that currently allows absolute quantification of the fat deposition in the liver, and it is being widely used to estimate hepatic steatosis as a substitute for biopsy. The test is done without intravenous contrast and has a total duration of about 10 minutes.

5) Biochemical tests: fasting blood will be drawn to measure glucose, insulin, lipid profile and liver enzymes, in addition to some hormones related to obesity (leptin).

6) The physical condition will be evaluated through physical tests in a gym with a duration of approximately 1 hour and a half.

7) We will put an accelerometer, which is a device that records movement and that you will wear with a strap on your hip, for 7 days. It is absolutely harmless.

8) We will give you questionnaires that you can fill out sometimes at home and sometimes with us during the tests, about dietary habits, psychological health and lifestyles in general.

9) We will weigh and size the boys and girls monthly to monitor growth and changes in weight.

ARE THERE ANY RISKS?

During the stress test, certain changes can occur in the body, including changes in blood pressure. Every effort will be made to minimize potential risks by evaluating preliminary information concerning your health and physical condition, and by observations made during the test. During the test there will be a doctor.

Drawing blood could lead to a bruise.

In DEXA scans to study body composition, X-rays will be used, so your child will receive a minimal dose of radiation (one-tenth of that received in a chest x-ray).

WHAT ARE THE BENEFITS?

We hope that children reduce their cardiovascular risk and improve weight and body composition. In addition, after carrying out the different tests and the physical exercise intervention program, the participants and their parents or guardians will be able to have information about their state of health, general physical condition and their evolution and changes produced at the end of the intervention period. Of the investigation. On the other hand, if we detect any anomaly in any of the tests carried out (blood tests, blood pressure, MRI, etc.), the parents or guardians will be informed immediately and a report will be prepared for the pediatrician.

Your child may be randomly involved in the control group. In that case, you will undergo the same tests as the rest so you will have the same information about your

health. As we understand that participating in the physical exercise program has benefits for the physical and psychological health of children, once the 6-month period is over, they will be offered the same physical exercise program as the “treated” group, without repeating the tests.

WHO IS PARTICIPATING IN THIS STUDY?

160 boys and girls from Vitoria-Gasteiz with ages between 9 and 11 and who are overweight or obese participated in the study.

It is important that you understand that participation in this study is voluntary and that you have the right to abstain from participating at any time, without prejudice to the assistance your child receives. If you decide to have your child participate, it is necessary that the father or mother (or legal guardian) sign the informed consent that we enclose.

SAMPLE CONSERVATION AND DATA PROTECTION

It is planned that the blood samples will be kept frozen and duly coded and anonymized at the LEIA-Tecnalia Research Unit located at the Txagorritxu Hospital (HUA). In this way, the analyzes could be repeated if necessary and always with the purpose and objectives described in this project. In any case, if you so wish, we will proceed to destroy the samples once the investigation is completed.

The data will be protected from unauthorized uses by persons outside the investigation and their confidentiality will be respected in accordance with Organic Law 15/1999, of December 13, on the Protection of Personal Data and Law 41 / 2002, of November 14, basic law regulating patient autonomy and rights and obligations regarding information and clinical documentation. Therefore, the information generated in this study will be considered strictly confidential between the participating parties, allowing its inspection, where appropriate, by the Health Authorities. The person responsible for data processing is the Principal Investigator, Idoia Labayen.

FINAL CLAUSE:

The parents or legal guardians of the participant can expand the information about this study at any time and solve any doubts as well as obtain the results of their participation in the project by contacting the director of the same at the email address or telephone that appears at the bottom of the page.

Principal investigator: Idoia Labayen (945014346)

Email: idoia.labayen@ehu.es

INFORMED CONSENT

"EFIGRO" RESEARCH PROJECT:
"Effects of a physical exercise program on liver fat in overweight boys and girls"

Mss./Mr. ____

as the parent / legal guardian of (check all that apply)

ID: ____

You have freely chosen to have your child participate in the research explained above.

Confidentiality of the results: All the personal data that you have provided us for this investigation is confidential. We will protect them as required by law and will only use them for research that we have explained to you, although this may require us to send them to other research groups that collaborate with us. In that case, we would never send them the name or any information that could identify the child. If you want to consult or modify them, or that we eliminate them or not use them for any of the purposes of the investigation, please contact us at the following address:

Principal investigator: Idoia Labayen (945014346)

Email: idoia.labayen@ehu.es

Important information:

1. The participant and her parents or legal guardians are informed that they can freely revoke her consent in writing at any time, and that this will not affect the usual care with their specialists.

2. The participant and her parents or legal guardians are informed of their right to be given a copy of the signed document and of the results obtained from her participation in the study.

3. The participant and her parents or legal guardians are informed that she should not make any changes in participation in extracurricular sports while participating in the project.

I have had detailed information about the tests your child will take to participate in this research. I have understood the potential risks and benefits of testing, and that I am completely free to choose to have my child drop testing at any time.

Signature: ....… Date:

Signature of the principal investigator:

FINAL CLAUSE: The parents or legal guardians of the participant can expand the information about this study at any time and solve any doubt as well as obtain the results of their participation in the project by contacting the director of the same at the email address email or telephone number listed at the bottom of the page.

ETHIC COMMITTEE APPROVAL

CEIC E
Euskadiko Ikerketa Klinikoetarako Batzorde Etikoa
Comité Ético de Investigación Clínica de Euskadi

EUSKO JAURLARITZA
GOBIERNO VASCO
OSASUN SAILA
DEPARTAMENTO DE SALUD

INFORME DEL COMITE ETICO DE INVESTIGACION CLINICA DE EUSKADI (CEIC-E)

Dra. Iciar Alfonso Farnós como Vicepresidenta del CEIC de la Comunidad Autónoma de País Vasco (CEIC-E)

CERTIFICA

Que este Comité, de acuerdo a la ley 14/2007 de Investigación Biomédica, Principios éticos de la declaración de Helsinki y resto de principios éticos aplicables, ha evaluado el proyecto de investigación, titulado **Efectos de un programa de ejercicio físico sobre la grasa hepática en niños y niñas con sobrepeso,** Código interno: PI2014045

Versión del Protocolo: versión 04 de Marzo de 2014
Versión de la HIP:
PADRES/TUTORES / Versión 2 de fecha 04.03.2014
RESONANCIA MAGNÉTICA ABDOMINAL: Versión 2, de 04/03/2014

Y que este Comité reunido el día 26/03/2014 (recogido en acta 04/2014) ha decidido emitir **dictamen favorable** a que dicho proyecto sea realizado por los siguientes investigadores:

- Idoia Labayen *Universidad del País Vasco UPV/EHU*

Lo que firmo en Vitoria, a 8 de abril de 2014

Fdo:

EUSKO JAURLARITZA
GOBIERNO VASCO
OSASUN SAILA
DEPARTAMENTO DE SALUD
10 ABR 2014
Euskadiko Ikerketa Klinikoetarako Batzorde Etikoa
Comité Ético de Investigación Clínica de Euskadi (CEIC-E)

Dra. Iciar Alfonso Farnós
Vicepresidenta del CEIC de la Comunidad Autónoma del País Vasco (CEIC-E)

TRANSLATION OF THE ETHIC COMMITTEE APPROVAL

Dr. Iciar Alfonso Famós as Vicedean of the CEI of the Basque Country (CEIC-E)

CERTIFIED

That this Committee, according to the law 14/2007 of Biomedical Research, Ethical Principles of the Declaration of Helsinki and the remaining ethical principles applied, has evaluated the research project titled “Effects of a physical exercise program on liver fat in overweight boys and girls”, internal code: PI2014045

Protocol version: March 4th 2014

Participant informative sheet: PARENTS/LEGAL TUTORS / Version 2: March 4th 2014

Abdominal magnetic resonance imaging: Version 2 of March 4th 2014.

And that Committee met on 26/03/2014 (recorded in the minutes 04/2014) has decided issue a favourable opinion of performing this project for the following researchers:

- Idoia Labayen, Basque Country University UPV/EHU

Signed in Vitoria, 8th April 2014

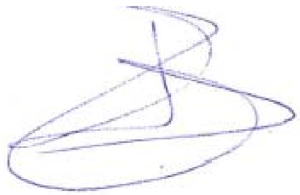

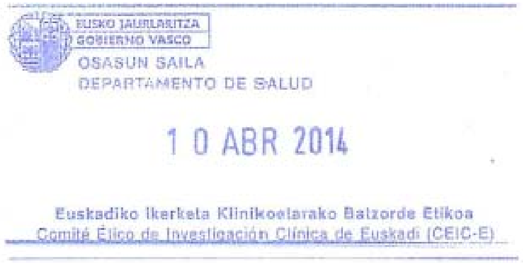

Dr. Iciar Alfonso Farnós

Vicedean of the CEI of the Basque Country (CEIC-E)
