# Supplementary material for: Effects of a Family-Based Lifestyle Intervention Plus Supervised Exercise Training on Abdominal Fat Depots in Children With Overweight or Obesity: A Secondary Analysis of a Nonrandomized Clinical Trial
Source: JAMA Netw Open. 2022 Nov 28;5(11):e2243864. doi: 10.1001/jamanetworkopen.2022.43864 (PMC9706365; doi:10.1001/jamanetworkopen.2022.43864)
Supplement: Supplement 2. — eMethods. Family-Based Lifestyle and Psychoeducation Program, Exercise Intervention Program, and Data Analysis eReferences eTable 1. Subject Characteristics eTable 2. Abdominal Visceral, Subcutaneous, and Intermuscular Adipose Tissue From 3 Axial Sections at Baseline and After the Intervention (Per-Protocol Analysis) eTable 3. Abdominal Visceral and Subcutaneous Adipose Tissue, Intermuscular Adipose Tissue, and Pancreatic Adipose Tissue at Baseline and After the Intervention (Per-Protocol Analysis Additionally Including a Minimum of 50% of Attendance of the Exercise Sessions) eTable 4. Abdominal Visceral and Subcutaneous Adipose Tissue, Intermuscular Adipose Tissue, and Pancreatic Adipose Tissue at Baseline and After the Intervention (Intention-to-Treat Analyses) eTable 5. Abdominal Visceral and Subcutaneous Adipose Tissue, Intermuscular Adipose Tissue, and Pancreatic Adipose Tissue at Baseline and After the Intervention (Per-Protocol Analysis Using Only Those Children and Parents Who Were Randomized) eFigure 1. Changes in Abdominal Visceral Adipose Tissue and Abdominal Subcutaneous Adipose Tissue in Participants in the Lifestyle and Psychoeducational Program (Control Group) and the Same Program Plus Supervised Exercise Training (Exercise Group) eFigure 2. Mediation Model to Determine Whether Changes in VAT Area Mediated Changes in Insulin Resistance (HOMA) eFigure 3. Changes in Abdominal Visceral Adipose Tissue in Participants in the Lifestyle and Psychoeducation Program (Control Group) and in the Same Program Plus Supervised Exercise Training (Exercise Group) in Intention-to-Treat Analyses eFigure 4. Changes in Abdominal Subcutaneous Adipose Tissue, Intermuscular Abdominal, and Pancreatic Fat Fraction in Participants in the Lifestyle and Psychoeducation Program (Control Group) and in the Same Program Plus Supervised Exercise Training (Exercise Group) in Intention-to-Treat Analyses eFigure 5. Mediation Model to Determine Whether Changes in VAT Area Mediated Changes [file jamanetwopen-e2243864-s002.pdf]

Supplementary Online Content

Cadenas-Sanchez C, Cabeza R, Idoate F, et al. Effects of a family-based lifestyle intervention plus supervised exercise training on abdominal fat depots in children with overweight or obesity: a secondary analysis of a nonrandomized clinical trial. *JAMA Netw Open*. 2022;5(11):e2243864. doi:10.1001/jamanetworkopen.2022.43864

eMethods. Family-Based Lifestyle and Psychoeducation Program, Exercise Intervention Program, and Data Analysis
eReferences
eTable 1. Subject Characteristics
eTable 2. Abdominal Visceral, Subcutaneous, and Intermuscular Adipose Tissue From 3 Axial Sections at Baseline and After the Intervention (Per-Protocol Analysis)
eTable 3. Abdominal Visceral and Subcutaneous Adipose Tissue, Intermuscular Adipose Tissue, and Pancreatic Adipose Tissue at Baseline and After the Intervention (Per-Protocol Analysis Additionally Including a Minimum of 50% of Attendance of the Exercise Sessions)
eTable 4. Abdominal Visceral and Subcutaneous Adipose Tissue, Intermuscular Adipose Tissue, and Pancreatic Adipose Tissue at Baseline and After the Intervention (Intention-to-Treat Analyses)
eTable 5. Abdominal Visceral and Subcutaneous Adipose Tissue, Intermuscular Adipose Tissue, and Pancreatic Adipose Tissue at Baseline and After the Intervention (Per-Protocol Analysis Using Only Those Children and Parents Who Were Randomized)
eFigure 1. Changes in Abdominal Visceral Adipose Tissue and Abdominal Subcutaneous Adipose Tissue in Participants in the Lifestyle and Psychoeducational Program (Control Group) and the Same Program Plus Supervised Exercise Training (Exercise Group)
eFigure 2. Mediation Model to Determine Whether Changes in VAT Area Mediated Changes in Insulin Resistance (HOMA)
eFigure 3. Changes in Abdominal Visceral Adipose Tissue in Participants in the Lifestyle and Psychoeducation Program (Control Group) and in the Same Program Plus Supervised Exercise Training (Exercise Group) in Intention-to-Treat Analyses
eFigure 4. Changes in Abdominal Subcutaneous Adipose Tissue, Intermuscular Abdominal, and Pancreatic Fat Fraction in Participants in the Lifestyle and Psychoeducation Program (Control Group) and in the Same Program Plus Supervised Exercise Training (Exercise Group) in Intention-to-Treat Analyses
eFigure 5. Mediation Model to Determine Whether Changes in VAT Area Mediated Changes in Insulin Resistance (HOMA) in Intention-to-Treat Analyses
eFigure 6. Mediation Model to Determine Whether Changes in VAT Area Mediated Changes in Insulin Resistance (HOMA) in Sensitivity Analysis Using Only Those Children Who Were Randomized

This supplementary material has been provided by the authors to give readers additional information about their work.

© 2022 Cadenas-Sanchez C et al. *JAMA Network Open*.

eMethods. Family-Based Lifestyle and Psychoeducation Program, Exercise Intervention Program, and Data Analysis

Family-based lifestyle and psychoeducation program

The program included two sessions/month. The sessions were delivered to both parents (or caregivers) and children, separately. Being aware that the intervention program demands time to the families and children, and thus, this could affect the adherence to the intervention, the healthy lifestyle and psychoeducational interventions were developed simultaneously. In this regard, families came to the facilities of the Faculty of Physical Activity and Sport Sciences of the University of the Basque Country, 11 times (sessions) for 90 minutes, over the 22-week intervention. Children attended first to the lifestyle education program (45 min/session) while their parents or caregivers were participating in the psychoeducation program (45 min/session). After finishing each session, children received the psychoeducation program whilst their parents or caregivers received the lifestyle education.

The lifestyle intervention program focused on promoting changes in three lifestyle behaviors areas known as the most relevant lifestyle-related risk for obesity and its co-morbidities: diet, physical activity, and stress. The aim of the program was to increase children's and parent's self-efficacy, knowledge, and motivation to adopt healthier dietary habits, increase physical activity levels, and reduce sedentary behavior. To achieve these objectives, the intervention program included 11 sessions (**Table 1**) combining different aspects of healthy behaviours with the help of teaching materials such as photographs, power point slides, stories, games such as crosswords, riddles, discussions, etc., and three workshops designed by research nutritionists for children

© 2022 Cadenas-Sanchez C et al. *JAMA Network Open*.

and their parents or caregivers. Key messages were consistently included across all sessions. Moreover, written information emphasizing key messages will be given to parents or caregivers to take home after the workshops. To reinforce the messages delivered through the sessions, and to involve parents or caregivers on lifestyle behavior changes, we provided them matching homework activities. Likewise, children had a booklet with 11 chapters (one per lesson), containing three objectives to follow every day. The content, doubts, and comments of the homework activities were discussed in the next session.

Table 1. Objectives and Topics of the Healthy Lifestyle Education Program

	Topics and aims	Type of session
First session	To learn how to classify foods according to their sugar and fat content	Lesson
Second session	To promote the consumption of a healthy and complete breakfast understanding its importance for health	Lesson
Third session	To promote the increase in daily physical activity level up to 30 min/day and the reduction of time spent on sedentary behaviours such as TV viewing, computer and video games, and smart phones explaining why this is important	Lesson
Fourth session	a) To learn about the importance of eating five times a day for health and body mass control<br>b) To learn about healthy options for morning and afternoon snacks	Lesson
Fifth session	To enhance daily consumption of fruits and vegetables	Workshop:<br>healthy cooking workshop to prepare a common recipe
Sixth session	To reduce the consumption of energy dense foods and sugar sweetened drinks	Workshop:<br>To weigh the sugar content of some foods and beverages typically consumed by the children
Seventh session	To promote the increase in daily physical activity level up to 60 min/day and the reduction of time spent on sedentary behaviours such as TV viewing, computer and video games, and smart phones	Lesson
Eighth session	To distinguish between hunger and appetite<br>to enhance awareness of the importance of an adequate sleep duration	Lesson

© 2022 Cadenas-Sanchez C et al. *JAMA Network Open*.

Ninth session	To learn and understand the nutritional information of food labelling	Lesson
Tenth session	To clarify certain popular myths related to some foods	Lesson
Eleventh session	To practice and integrate the knowledge acquired	Workshop:<br>to develop complete and healthy menus

The psychoeducation program for parents or caregivers aimed to: i) increase awareness of the problems that could derive from children with overweight or obesity, ii) provide parental skills to get favourable family environment in order to make positive changes in their lifestyles; and iii) learn assertive communication skills. For reaching these objectives, the intervention program consisted of sessions where different topics were presented and discussed (e.g., teasing at school and how to deal with this problem, expressing emotions and educating their children in managing them or changing habits in the family context). For children, the psychoeducation program aimed to: i) ease tools to manage emotions and feelings that they experience because of their condition of being overweight or obese, and ii) to provide skills to improve their self-esteem and the psychological and social well-being. In order to reach these objectives, the intervention program consisted of sessions where different topics were presented and discussed (e.g., teasing at school, putting into words the emotions and feelings that my body makes me feel or activities such as expressing parts of the body that each one likes of herself/himself). All sessions were designed and taught by psychologist.

Exercise intervention program

This program involved three exercise sessions/week (minimum recommended). The sessions were designed and supervised by trainers with university-level sports science education, and held at the training facilities of the Faculty of Physical Activity and Sport Sciences, University of the Basque Country (Vitoria-Gasteiz, Spain). The sessions

© 2022 Cadenas-Sanchez C et al. *JAMA Network Open*.

were divided into three parts: i) warm-up (10 min), ii) aerobic and resistance training (70 min), and iii) cool-down (5-10 min).

i) Warm-up consisted of progressively increasing the heart rate through the playing of two games (5 min each of either running in pairs, running individually, playing tag, etc.).
ii) The aerobic and resistance training made up the main part of each session. Aerobic training was mainly multigame-based exercises (five games of 12 minutes each, e.g., sharks and minnows, red light-green light, relays, sack races, or playing baseball, handball, or basketball). Resistance training involved nine muscular strength exercises focused on self-loading training, and exercising in pairs using medicine balls, fitballs, therabands, etc. The exercises performed included biceps curls, bench dips (triceps), triceps extension with therabands, band pull-aparts, lateral abdominal muscle workouts, medicine ball abdominal muscle workouts, medicine ball throw-downs, fitball bridges, standard and lateral planks, and lung and squat exercises, etc.
iii) Cooling down consisted of full body stretching (biceps, triceps, shoulder, neck, hip, lower back, gluteus, abductor, quadriceps, hamstrings, and gastrocnemius) and relaxing exercises (e.g., breath focus exercise, mindfulness, etc.).

The main part of the exercise session promoted moderate-to-vigorous aerobic exercise. Individual exercise intensity was calculated from ventilatory thresholds[1] and the maximum heart rate as determined via a cardiopulmonary exercise on a treadmill ergometer with respiratory gas analysis[2]. The training sessions were designed to progressively increase the intensity of the aerobic exercises over the 22-week period. During the sessions, subjects were monitored using a Polar RS300X heart rate monitor

© 2022 Cadenas-Sanchez C et al. *JAMA Network Open*.

(Kempele, Finland) to ensure the target heart rate was reached. Heart rate monitors were programmed according to individual ventilatory thresholds and the maximum heart rate.

Magnetic resonance imaging: screening analysis information

All images were acquired using a 1.5 T Siemens Magnetom Avanto system (Siemens Medical Solutions, Germany) equipped with a phased-array surface coil and a spine array coil, and employing the proprietor's Work-in-Progress software package (version syngo.MR B17A). A three-dimensional two-point Dixon sequence was used under breath-holding conditions to obtain two-point opposed- and in-phase data with Dixon water/fat separation. The acquisition time was 17s, the repetition time (TR) 7.46 ms, echo time (TE) 2.38/4.76 ms, flip angle 10º, field of view 420 mm, and the acquisition matrix 256 × 256 for 60 slices for abdominal outcomes, and 160 × 160 for 60 for pancreatic fat (resolution 1.64 × 1.64 × 5 mm). Images were obtained with subjects in the supine position. All stacks were acquired with breath-holding. Sagittal, coronal and transverse abdominal localizers (from the diaphragm to the symphysis pubis) were used to determine the precise location of each image with respect to the vertebral discs.

A 2-point Dixon gradient-echo pulse sequence was used to separate the tissue water signal from the lipid signal [3]. Four images were thus obtained: i) water-only images (SIW), fat-only images (SIF), in-phase images (water *plus* fat), and out-of-phase images (water *minus* fat) (**Figure 1**).

© 2022 Cadenas-Sanchez C et al. *JAMA Network Open*.

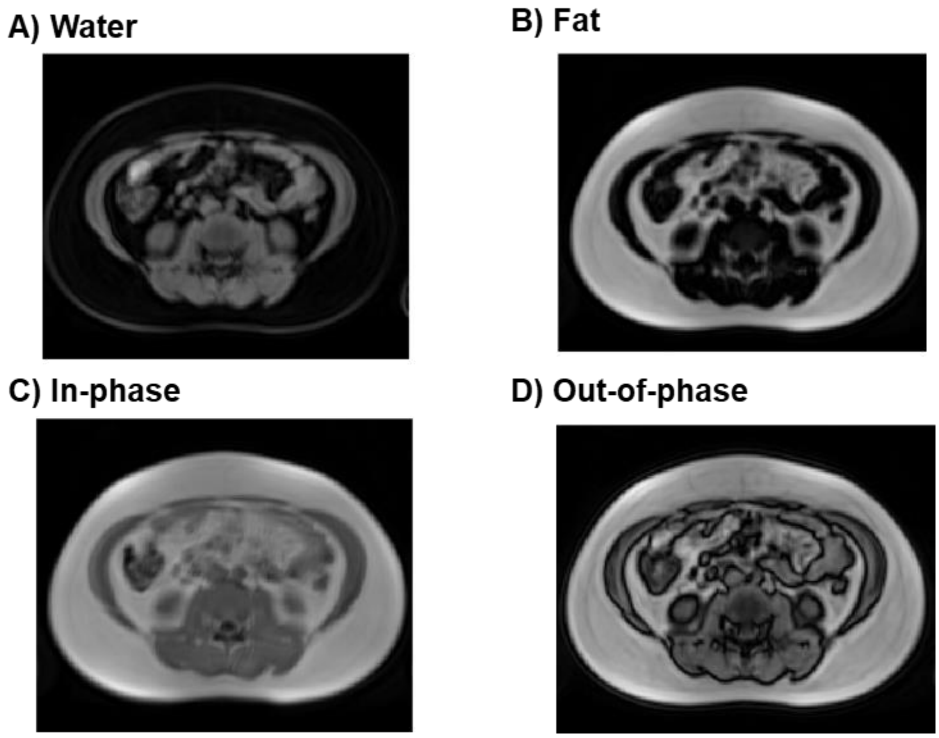

Figure 1. Dixon gradient-echo pulse sequence separation: water-only image (panel A), fat-only image (panel B), in-phase image (panel C), and out-of-phase image (panel D). Visceral (VAT), abdominal subcutaneous (ASAT), intermuscular abdominal (IMAAT), and pancreatic adipose tissue (PAT) were analyzed separately.

Abdominal adipose tissues: visceral, subcutaneous, and intermuscular.

A MATLAB-based semi-automatic segmentation algorithm designed at our laboratory was used in all analyses.

Two additional images were produced from the four MRI images: 1) the fat fraction image, or parametric image, calculated[4] as the (SIF) / (SIF + SIW), and 2) the 2F – W image, calculated as: 2F – W = 2 * SIF – SIW (where F is the image of the fat, and W the image of the water).

It is important to highlight that we could not perform image analysis with the multiple image approach since it elevated cost. Nevertheless, in the last decades, the scientific evidence elucidates that a single image is often chosen to approximate total volumes

© 2022 Cadenas-Sanchez C et al. *JAMA Network Open*.

and thus, it was sufficient for prediction[5–9]. Therefore, the selection of the area of the broad set of fat depots examined in this study provides a reliable and fast estimation of its volume.

For abdominal tissue classification:

The Otsu thresholding algorithm was used to separate the body area from the background[10] of the MRI image. This algorithm calculates a threshold intensity value based on the maximization of the interclass variance. By combining the 2F-W image and the mask obtained from the thresholding, the abdomen and arms were detected as regions of interest (ROIs - (with the abdomen the largest)). In this manner, the external limit of the abdomen can be easily identified as the boundary pixels of this region.

1. A researcher experienced in this type of analysis made a coarse delineation of the abdominal visceral area (avoiding the muscle tissue). The active contours algorithm[11] was then applied to this annotation using the 2F – W image. The coarse contour sketched by the researcher was fitted to the limits between the abdominal viscera area and the internal wall of the abdominal muscles. As result of this step, the most external part of the abdomen was identified.
2. Lean tissue was easily identified by means of the Otsu algorithm. The darkest region (tissue water) was tagged as an abdominal musculature. A more accurate delineation of the external contour of the musculature was then calculated by means of active contours. As result, the ASAT internal border, which constitutes the external wall of the abdominal musculature, was found.

As a result of these steps, the abdominal viscera compartment, ASAT area, and abdominal muscle area were all identified and labeled.

© 2022 Cadenas-Sanchez C et al. *JAMA Network Open*.

3. The non-supervised clustering (K-means) method was used to identify fat, muscle and visceral tissues, and the abdominal viscera compartment[12,13] in the fat fraction image. This type of algorithm classifies voxel intensity values into a predefined number of classes. In this work, three classes were selected: water, air and fat. As a result, average voxel intensity values were obtained as identifiers for each class. The lowest intensity value corresponded to fat voxels while tissue water corresponds to the highest. The remaining voxels, were identified as representing air.

   The intensity values obtained with the K-means algorithm were then used to calculate further segmentations:

   - The adipose tissue inside the abdominal viscera area was categorized as VAT.
   - The adipose tissue between the muscular external border and the visceral area wall was categorized as IMAAT.
   - The tissues identified as water in the visceral compartment area were classified as viscera.
   - The tissues identified as water within the abdominal musculature area (the area between the muscular external wall and the abdominal visceral limit) was classified as muscle tissue.

   Figure 2 shows the results of the segmentation identifying the different tissues.

© 2022 Cadenas-Sanchez C et al. *JAMA Network Open*.

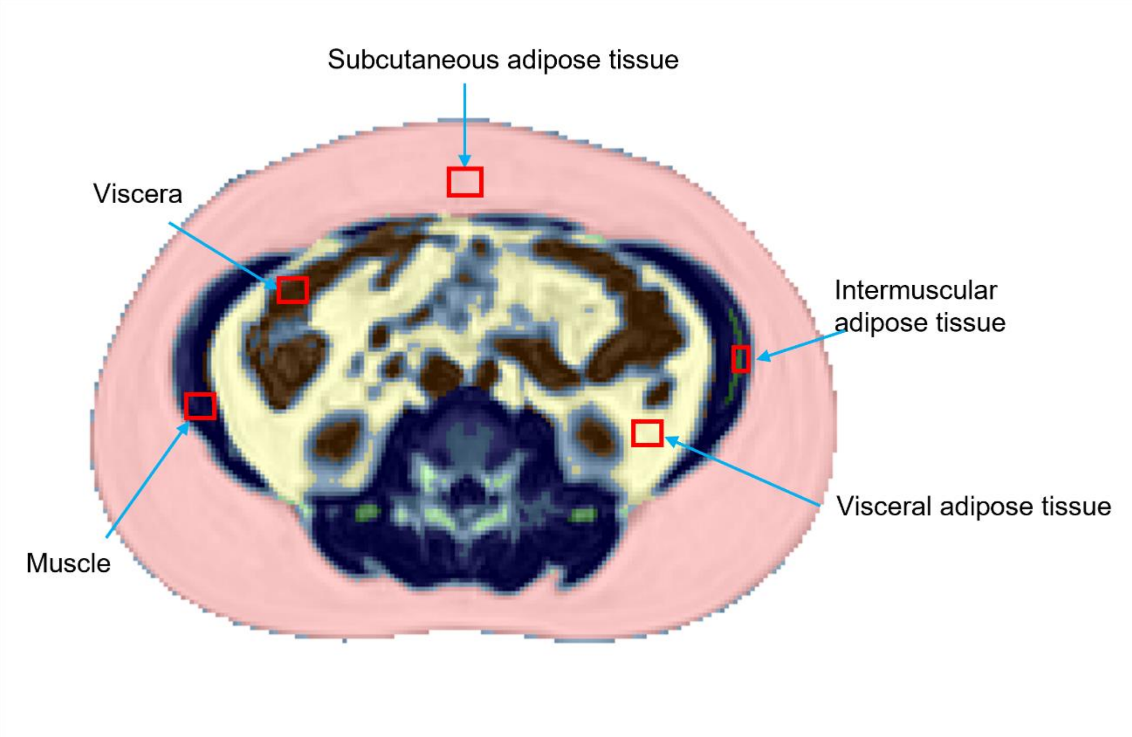

Figure 2. Magnetic resonance imaging segmentation. Abdominal adipose tissues (visceral, subcutaneous, and intermuscular), viscera and muscle are shown.

Once the image had been divided into its constitutive regions, the following were calculated:

- The mean fat fraction for VAT and ASAT (using the fat fraction image).
- The area (cm^2) of VAT and ASAT in each image was determined by summing the pixels in each of these regions and multiplying by the individual pixel area.
- The total sectional area.

All analyses were performed using a validated semiautomatic tool; the statistical results show it to be comparable to the gold-standard manual sliceOmatic segmentation method.

Pancreatic adipose tissue

To quantify PAT, the 3D six-echo gradient sequences were analyzed, using OsiriX v. 6.0 software (Pixmeo Sarl, Switzerland), by a radiologist with 20 years of experience.

© 2022 Cadenas-Sanchez C et al. *JAMA Network Open*.

Three ROIs (of approximately 50 mm^2) were drawn manually as previously reported[14,15]. These ROIs include the periductal and marginal pancreatic parenchyma. For both the analysis of the whole image and of the individual slices, the surrounding extra-pancreatic adipose tissue and vessels were carefully avoid. The water-suppressed images obtained were used to set the three ROIs at the head, body, and tail of the pancreas.

The ROIs were then used with the fat fraction maps. ROIs for post-intervention MRIs were carefully colocalized with the ROIs from the baseline MRI, and adjusted to include only pancreatic parenchyma.

To determine the mean PAT (fat fraction [%]), the mean fat fraction of all ROIs in each part of the pancreas (the head, body, and tail) was calculated. **Figure 3** shows a representative MRI highlighting the PAT.

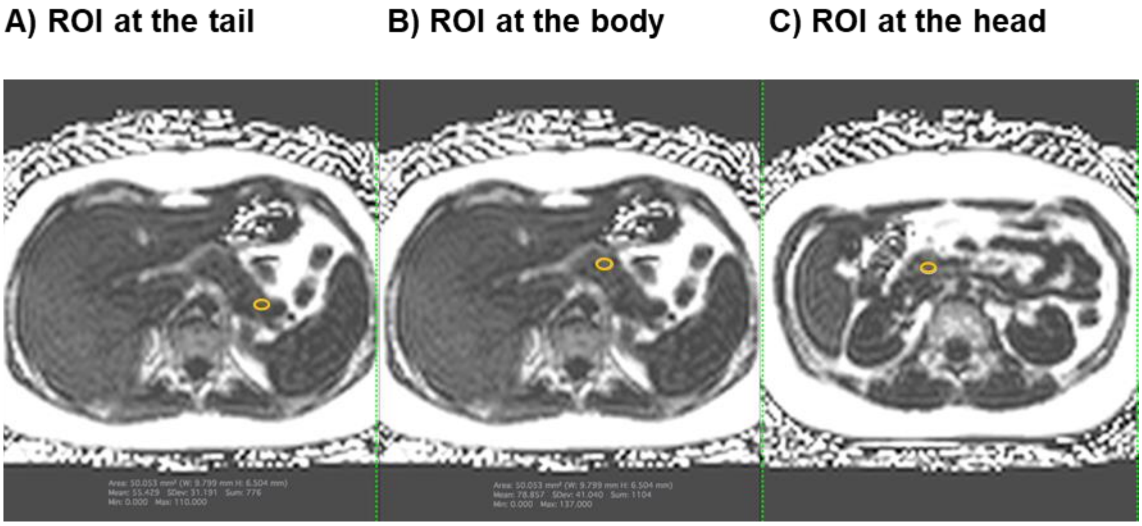

Figure 3. Mapping the pancreatic adipose tissue using a six-echo Dixon-based magnetic resonance sequence. The ROIs (represented by orange ovals) were located at the tail (panel A), body (panel B) and head (panel C) of the pancreas. The mean PAT fat fraction (%) values are shown in the boxes at the bottom of each image.

© 2022 Cadenas-Sanchez C et al. *JAMA Network Open*.

eReferences

1. Quinart S, Mourot L, Nègre V, et al. Ventilatory thresholds determined from HRV: comparison of 2 methods in obese adolescents. *Int J Sports Med*. 2014;35(3):203-208. doi:10.1055/s-0033-1345172

2. Medicine AC of S. *ACSM's Guidelines for Exercise Testing and Prescription*. Tenth Edit. Wolters Kluwer; 2017.

3. Dixon WT. Simple proton spectroscopic imaging. *Radiology*. 1984;153(1):189-194. doi:10.1148/radiology.153.1.6089263

4. Fischer MA, Pfirrmann CWA, Espinosa N, Raptis DA, Buck FM. Dixon-based MRI for assessment of muscle-fat content in phantoms, healthy volunteers and patients with achillodynia: comparison to visual assessment of calf muscle quality. *Eur Radiol*. 2014;24(6):1366-1375. doi:10.1007/s00330-014-3121-1

5. Abate N, Garg A, Coleman R, Grundy SM, Peshock RM. Prediction of total subcutaneous abdominal, intraperitoneal, and retroperitoneal adipose tissue masses in men by a single axial magnetic resonance imaging slice. *Am J Clin Nutr*. 1997;65(2):403-408. doi:10.1093/ajcn/65.2.403

6. Demerath EW, Shen W, Lee M, et al. Approximation of total visceral adipose tissue with a single magnetic resonance image. *Am J Clin Nutr*. 2007;85(2):362-368. doi:10.1093/ajcn/85.2.362

7. Han TS, Kelly IE, Walsh K, Greene RM, Lean ME. Relationship between volumes and areas from single transverse scans of intra-abdominal fat measured by magnetic resonance imaging. *Int J Obes Relat Metab Disord J Int Assoc Study Obes*. 1997;21(12):1161-1166. doi:10.1038/sj.ijo.0800530

8. Schwenzer NF, Machann J, Schraml C, et al. Quantitative analysis of adipose tissue in single transverse slices for estimation of volumes of relevant fat tissue compartments: a study in a large cohort of subjects at risk for type 2 diabetes by MRI with comparison to anthropometric data. *Invest Radiol*. 2010;45(12):788-794. doi:10.1097/RLI.0b013e3181f10fe1

9. Maislin G, Ahmed MM, Gooneratne N, et al. Single slice vs. volumetric MR assessment of visceral adipose tissue: reliability and validity among the overweight and obese. *Obesity (Silver Spring)*. 2012;20(10):2124-2132. doi:10.1038/oby.2012.53

10. Otsu N. A Threshold Selection Method from Gray-Level Histograms. *IEEE Trans Syst Man Cybern*. 1979;9(1):62-66. doi:10.1109/TSMC.1979.4310076

11. Kass M, Witkin A, Terzopoulos D. Snakes: Active contour models. *Int J Comput Vis*. 1988;1(4):321-331. doi:10.1007/BF00133570

12. Forgy E. Cluster analysis of multivariate data: efficiency versus interpretability of classifications. *Biometrics*. 1965;21(3):768-769.

13. SP L. Least Squares Quantization in PCM. *IEEE Trans Inf Theory*. 1982;28(2):129-137.

14. Chen Y, Long L, Jiang Z, Zhang L, Zhong D, Huang X. Quantification of

© 2022 Cadenas-Sanchez C et al. *JAMA Network Open*.

pancreatic proton density fat fraction in diabetic pigs using MR imaging and IDEAL-IQ sequence. *BMC Med Imaging*. 2019;19(1):38. doi:10.1186/s12880-019-0336-2

15. Covarrubias Y, Fowler KJ, Mamidipalli A, et al. Pilot study on longitudinal change in pancreatic proton density fat fraction during a weight-loss surgery program in adults with obesity. *J Magn Reson Imaging*. 2019;50(4):1092-1102. doi:10.1002/jmri.26671

© 2022 Cadenas-Sanchez C et al. *JAMA Network Open*.

eTable 1. Subject Characteristics

	Control group			Exercise group	
	N	Mean (SD)		N	Mean (SD)
Age (years)	57	10.6 (1.1)		59	10.5 (1.0)
Girls (n, %)	57	30 (52.6)		59	32 (54.2)
Mother's origin not Spanish (N, %)	57	6 (10.5)		59	13 (22.0)
High maternal educational level (N, %)	57	46 (80.7)		58	38 (65.5)
Family history of diabetes (N, %)	57	7 (12.3)		58	2 (3.4)
Tanner stage (N, %)					
Telarche or gonadarche	55			53	
I		20 (36.4)			22 (41.5)
II		20 (36.4)			12 (22.6)
III		12 (21.8)			11 (20.8)
IV-V		3 (5.5)			8 (15.1)
Pubarche	55			53	
I		20 (36.4)			17 (32.1)
II		22 (40.0)			20 (37.7)
III		7 (12.7)			10 (18.9)
IV-V		6 (10.9)			6 (11.3)
Body mass index (kg/m^2)	57	25.2 (2.7)		59	25.8 (3.7)
Waist circumference (cm)	57	78.1 (7.3)		59	79.7 (7.6)
Insulin (IU/mL)	57	11.7 (4.8)		58	12.6 (5.0)
Glucose (mg/dL)	56	85.3 (5.5)		58	85.5 (5.3)
HOMA	56	2.5 (1.1)		58	2.7 (1.2)
Cardiorespiratory fitness - lab (total completion time, min)	44	10.0 (2.8)		51	9.0 (2.3)

HOMA: Homeostasis model assessment; SD: Standard deviation. Values are presented as means and SD, unless otherwise indicated. The control group intervention consisted of two family-based lifestyle and psychoeducation sessions/month. The exercise group intervention consisted of the same plus 3 sessions/week of supervised high-intensity exercise.

© 2022 Cadenas-Sanchez C et al. *JAMA Network Open*.

eTable 2. Abdominal Visceral, Subcutaneous, and Intermuscular Adipose Tissue From 3 Axial Sections at Baseline and After the Intervention (Per-Protocol Analysis)

	Control group (N = 52)		Exercise group (N = 49)		Mean difference between groups (95% CI)[a]	P[b]	Effect size[c]
	Pre	Post	Pre	Post			
	Mean (SD)	Mean (SD)	Mean (SD)	Mean (SD)			
**Abdominal adipose tissue**							
Visceral adipose tissue (VAT)							
VAT L2-L3 (area, cm^2)	45.9 (25.1)	41.4 (26.1)	45.4 (20.9)	36.1 (19.4)	-4.8 (-8.6, -1.0)	**0.007**	0.49
Fat fraction VAT L2-L3 (fat fraction, %)	84.0 (3.2)	83.7 (3.6)	84.9 (3.0)	83.3 (3.1)	-1.3 (-2.0, -0.5)	**0.001**	0.67
VAT L3 (area, cm^2)	46.7 (23.0)	41.4 (23.0)	46.0 (20.2)	36.9 (18.6)	-3.7 (-7.8, 0.3)	**0.03**	0.36
Fat fraction VAT L3 (fat fraction, %)	84.5 (3.4)	84.1 (3.7)	85.4 (3.0)	83.3 (3.5)	-1.7 (-2.4, -1.0)	**<0.001**	0.93
VAT L4-L5 (area, cm^2)	41.1 (18.9)	39.7 (17.6)	41.7 (14.9)	36.0 (13.5)	-4.2 (-8.1, -0.3)	**0.02**	0.42
Fat fraction VAT L4-L5 (fat fraction, %)	87.7 (2.0)	87.3 (2.4)	88.0 (2.2)	87.1 (2.4)	-0.5 (-1.2,0.2)	0.15	0.30
Subcutaneous adipose tissue (ASAT)							
ASAT L2-L3 (area, cm^2)	183.2 (66.1)	179.9 (74.1)	208.1 (67.9)	185.9 (70.4)	-18.9 (-28.3, -9.4)	**<0.001**	0.77
Fat fraction ASAT L2-L3 (fat fraction, %)	92.6 (1.9)	92.1 (2.9)	93.3 (1.4)	92.3 (2.0)	-0.5 (-1.1, 0.1)	**0.01**	0.36
ASAT L3 (area, cm^2)	208.9 (70.7)	203.7 (80.2)	236.5 (74.1)	212.4 (80.1)	-18.9 (-30.1, -7.8)	**0.001**	0.66
Fat fraction ASAT L3 (fat fraction, %)	93.3 (1.5)	92.7 (2.6)	93.9 (1.1)	92.8 (1.8)	-0.4 (-1.0, 0.1)	**0.007**	0.32
ASAT L4-L5 (area, cm^2)	279.3 (78.6)	265.4 (86.0)	306.3 (79.5)	278.6 (90.4)	-13.8 (-26.5, -1.1)	**0.02**	0.42
Fat fraction ASAT L4-L5 (fat fraction, %)	94.3 (0.9)	93.8 (1.6)	94.6 (0.7)	93.7 (1.0)	-0.3 (-0.7, 0.1)	**0.04**	0.33
Intermuscular abdominal adipose tissue (IMAAT)							
Fat fraction IMAAT L2-L3 (fat fraction, %)	6.9 (1.1)	6.9 (1.2)	7.0 (1.0)	6.6 (0.8)	-0.3 (-0.6, -0.1)	**0.01**	0.46
Fat fraction IMAAT L3 (fat fraction, %)	6.9 (1.2)	6.6 (1.3)	6.9 (0.9)	6.4 (0.8)	-0.2 (-0.5, 0.1)	0.08	0.31
Fat fraction IMAAT L4-L5 (fat fraction, %)	7.3 (0.9)	7.0 (1.0)	7.3 (0.9)	6.8 (0.8)	-0.2 (-0.4, 0.1)	0.08	0.29

SD: Standard deviation; CI: Confidence interval.

[a] Calculated using the difference between-group (exercise *minus* control) of changes (post *minus* pre).

© 2022 Cadenas-Sanchez C et al. *JAMA Network Open*.

[b] P indicates a significant difference between changes in the control and exercise groups (one-way analysis of covariance). Analyses were adjusted for baseline values, age, and sex. except for VAT and ASAT area adjustments were also made changes in height.
[c] Effect size calculated by Cohen's d and interpreted as small (d=0.2), medium (d=0.5), or large (d=0.8).

© 2022 Cadenas-Sanchez C et al. *JAMA Network Open*.

eTable 3. Abdominal Visceral and Subcutaneous Adipose Tissue, Intermuscular Adipose Tissue, and Pancreatic Adipose Tissue at Baseline and After the Intervention (Per-Protocol Analysis Additionally Including a Minimum of 50% of Attendance of the Exercise Sessions)

	Control group (N=52)		Exercise group (N=45)		Mean difference between groups (95% CI)[a]	P[b]	Effect size[c]
	Pre	Post	Pre	Post			
	Mean (SD)	Mean (SD)	Mean (SD)	Mean (SD)			
**Abdominal adipose tissue**							
Visceral adipose tissue (VAT)							
Mean VAT (area, cm^2)	44.6 (21.5)	40.8 (21.4)	45.0 (18.2)	36.8 (16.7)	-4.5 (-7.9, -1.2)	**0.004**	0.54
Mean VAT (fat fraction, %)	85.4 (2.7)	85.0 (3.0)	86.2 (2.6)	84.6 (2.9)	-1.2 (-1.8, -0.6)	**<0.001**	0.79
Subcutaneous adipose tissue (ASAT)							
Mean ASAT (area, cm^2)	223.8 (70.8)	216.4 (79.6)	252.3 (74.6)	226.9 (81.9)	-17.9 (-28.8, -7.0)	**0.001**	0.66
Mean ASAT (fat fraction, %)	93.4 (1.3)	93.0 (1.9)	93.9 (1.0)	92.9 (1.6)	-0.5 (-0.9, -0.1)	**0.007**	0.37
Intermuscular abdominal adipose tissue (IMAAT)							
Mean IMAAT (fat fraction, %)	7.0 (1.0)	6.9 (1.1)	7.0 (0.9)	6.6 (0.8)	-0.3 (-0.5, -0.01)	**0.02**	0.41
Pancreatic adipose tissue (PAT)[d]							
Mean PAT (fat fraction, %)	3.2 (2.6)	2.9 (2.5)	3.3 (1.9)	2.6 (1.7)	-0.3 (-1.0, 0.3)	0.23	0.23

SD: Standard deviation; CI: Confidence interval.

Data presented are for *per protocol* analysis additionally including a minimum of 50% of attendance of the exercise group (n total = 97 participants; n =45 in the exercise group).

[a] Calculated using the difference between-group (exercise *minus* control) of changes (post *minus* pre).

[b] P indicates a significant difference between changes in the control and exercise groups (one-way analysis of covariance). Analyses were adjusted for baseline values, age, and sex. except for VAT and ASAT area adjustments were also made changes in height.

[c] Effect size calculated by Cohen's *d* and interpreted as small (d=0.2), medium (d=0.5), or large (d=0.8).

[d] For PAT, the sample size was reduced for the control group (N=50) and exercise group (N=44).

© 2022 Cadenas-Sanchez C et al. *JAMA Network Open*.

eTable 4. Abdominal Visceral and Subcutaneous Adipose Tissue, Intermuscular Adipose Tissue, and Pancreatic Adipose Tissue at Baseline and After the Intervention (Intention-to-Treat Analyses)

	Control group (N = 57)		Exercise group (N = 59)			Mean difference between groups (95% CI)[a]	P[b]	Effect size[c]
	Pre	Post	Pre	Post				
	Mean (SD)	Mean (SD)	Mean (SD)	Mean (SD)				
**Abdominal adipose tissue**								
Visceral adipose tissue (VAT)								
VAT L2-L3 (area, cm^2)	45.3 (24.3)	41.7 (25.2)	48.3 (23.4)	38.2 (22.0)		-6.5 (-9.9, -3.0)	**0.001**	0.68
Fat fraction VAT L2-L3 (fat fraction, %)	84.0 (3.2)	83.7 (3.5)	85.1 (3.0)	83.3 (3.1)		-1.5 (-2.2, -0.9)	**<0.001**	0.84
VAT L3 (area, cm^2)	46.5 (22.2)	41.5 (22.1)	49.1 (23.6)	39.2 (21.1)		-4.9 (-8.6, -1.2)	**0.01**	0.49
Fat fraction VAT L3 (fat fraction, %)	84.5 (3.3)	84.2 (3.6)	85.6 (3.1)	83.4 (3.5)		-1.8 (-2.5, -1.2)	**<0.001**	1.04
VAT L4-L5 (area, cm^2)	40.8 (18.3)	40.0 (16.9)	43.4 (16.1)	37.0 (14.0)		-5.5 (-9.0, -1.9)	**0.004**	0.57
Fat fraction VAT L4-L5 (fat fraction, %)	87.7 (1.9)	87.4 (2.4)	88.1 (2.2)	87.2 (2.3)		-0.7 (-1.3, -0.1)	**0.04**	0.40
Mean VAT (area, cm^2)	44.2 (20.8)	41.1 (20.6)	46.9 (20.1)	38.2 (18.2)		-5.6 (-8.6, -2.7)	**<0.001**	0.71
Mean VAT fat fraction (fat fraction, %)	85.4 (2.6)	85.1 (3.0)	86.3 (2.6)	84.6 (2.8)		-1.3 (-1.9, -0.8)	**<0.001**	0.92
Subcutaneous adipose tissue (ASAT)								
ASAT L2-L3 (area, cm^2)	184.1 (65.1)	181.8 (73.0)	210.9 (73.8)	188.7 (76.0)		-19.9 (-28.3, -11.5)	**<0.001**	0.87
Fat fraction ASAT L2-L3 (fat fraction, %)	92.6 (1.8)	92.1 (2.9)	93.4 (1.4)	92.3 (2.0)		-0.5 (-1.0, -0.04)	**0.004**	0.40
ASAT L3 (area, cm^2)	210.2 (69.9)	206.3 (79.3)	239.9 (79.5)	215.3 (85.1)		-20.7 (-30.5, -10.9)	**<0.001**	0.78
Fat fraction ASAT L3 (fat fraction, %)	93.3 (1.5)	92.8 (2.6)	93.9 (1.1)	92.9 (1.8)		-0.5 (-1.0, -0.01)	**0.001**	0.37
ASAT L4-L5 (area, cm^2)	282.0 (79.9)	269.2 (87.3)	308.1 (84.4)	280.7 (94.0)		-14.6 (-25.8, -3.4)	**0.007**	0.48
Fat fraction ASAT L4-L5 (fat fraction, %)	94.3 (0.9)	93.8 (1.6)	94.6 (0.7)	93.8 (1.0)		-0.4 (-0.7, -0.02)	**0.01**	0.39
Mean ASAT (area, cm^2)	225.5 (70.6)	219.1 (79.3)	253.0 (78.4)	228.2 (84.4)		-18.4 (-27.7, -9.1)	**<0.001**	0.73
Mean ASAT fat fraction (fat fraction, %)	93.4 (1.3)	92.9 (2.3)	94.0 (1.0)	93.0 (1.6)		-0.5 (-0.9, -0.1)	**0.001**	0.40
Intermuscular abdominal adipose tissue (IMAAT)								
Fat fraction IMAAT L2-L3 (fat fraction, %)	7.0 (1.1)	6.9 (1.2)	7.2 (1.1)	6.8 (1.0)		-0.3 (-0.5, -0.1)	**0.01**	0.47

© 2022 Cadenas-Sanchez C et al. *JAMA Network Open*.

Fat fraction IMAAT L3 (fat fraction, %)	6.9 (1.2)	6.7 (1.2)	7.0 (1.1)	6.6 (1.0)		-0.2 (-0.4, 0.1)	0.24	0.24
Fat fraction IMAAT L4-L5 (fat fraction, %)	7.3 (0.9)	7.0 (0.9)	7.3 (0.9)	6.9 (0.8)		-0.2 (-0.4, 0.1)	0.18	0.24
Mean IMAAT fat fraction (fat fraction, %)	7.0 (1.0)	6.9 (1.1)	7.2 (0.9)	6.8 (0.9)		-0.2 (-0.41, 0.00)	0.05	0.37
Pancreatic adipose tissue (PAT)								
Mean PAT (fat fraction, %)	3.1 (2.6)	2.8 (2.4)	3.1 (1.7)	2.6 (1.6)		-0.2 (-0.70, 0.29)	0.35	0.15

SD: Standard deviation; CI: Confidence interval.

[a] Calculated using the difference between-group (exercise *minus* control) of changes (post *minus* pre).

[b] P indicates a significant difference between changes in the control and exercise groups (one-way analysis of covariance). Analyses were adjusted for baseline values, age, and sex, except for differences in VAT and ASAT area adjustments were also made changes in height.

[c] Effect size calculated by Cohen's d and interpreted as small (d=0.2), medium (d=0.5), or large (d=0.8).

© 2022 Cadenas-Sanchez C et al. *JAMA Network Open*.

eTable 5. Abdominal Visceral and Subcutaneous Adipose Tissue, Intermuscular Adipose Tissue, and Pancreatic Adipose Tissue at Baseline and After the Intervention (Per-Protocol Analysis Using Only Those Children and Parents Who Were Randomized)

	Control group (N=41)		Exercise group (N=49)		Mean difference between groups (95% CI)[a]	P[b]	Effect size[c]
	Pre	Post	Pre	Post			
	Mean (SD)	Mean (SD)	Mean (SD)	Mean (SD)			
**Abdominal adipose tissue**							
Visceral adipose tissue (VAT)							
Mean VAT (area, cm^2)	44.3 (20.3)	40.7 (21.1)	44.3 (17.7)	36.3 (16.3)	-4.4 (-7.8, -0.9)	**0.004**	0.53
Mean VAT (fat fraction, %)	85.4 (2.6)	85.1 (3.0)	86.1 (2.6)	84.5 (2.8)	-1.2 (-1.8, -0.5)	**<0.001**	0.75
Subcutaneous adipose tissue (ASAT)							
Mean ASAT (area, cm^2)	223.5 (71.6)	217.2 (81.4)	250.3 (72.8)	225.6 (79.6)	-17.4 (-28.9, -6.0)	**0.002**	0.64
Mean ASAT (fat fraction, %)	93.4 (1.4)	92.9 (2.4)	93.9 (1.0)	92.9 (1.6)	-0.4 (-0.9, -0.1)	**0.01**	0.35
Intermuscular abdominal adipose tissue (IMAAT)							
Mean IMAAT (fat fraction, %)	7.1 (1.1)	6.9 (1.1)	7.0 (0.8)	6.6 (0.7)	-0.3 (-0.5, -0.01)	**0.01**	0.44
Pancreatic adipose tissue (PAT)[d]							
Mean PAT (fat fraction, %)	3.1 (2.7)	2.8 (2.5)	3.2 (1.8)	2.6 (1.7)	-0.3 (-0.9, 0.3)	0.19	0.31

SD: Standard deviation; CI: Confidence interval.

Data are shown excluding those children/parents who were not randomized (n=11, all of them from the control group); therefore, only those randomized participants were included in the analyses.

[a] Calculated using the difference between-group (exercise *minus* control) of changes (post *minus* pre).

[b] P indicates a significant difference between changes in the control and exercise groups (one-way analysis of covariance). Analyses were adjusted for baseline values, age, and sex. except for VAT and ASAT area adjustments were also made changes in height.

[c] Effect size calculated by Cohen's *d* and interpreted as small (d=0.2), medium (d=0.5), or large (d=0.8).

[d] For PAT, the sample size was reduced for the control group (N=39) and exercise group (N=48).

© 2022 Cadenas-Sanchez C et al. *JAMA Network Open*.

eFigure 1. Changes in Abdominal Visceral Adipose Tissue and Abdominal Subcutaneous Adipose Tissue in Participants in the Lifestyle and Psychoeducational Program (Control Group) and the Same Program Plus Supervised Exercise Training (Exercise Group)

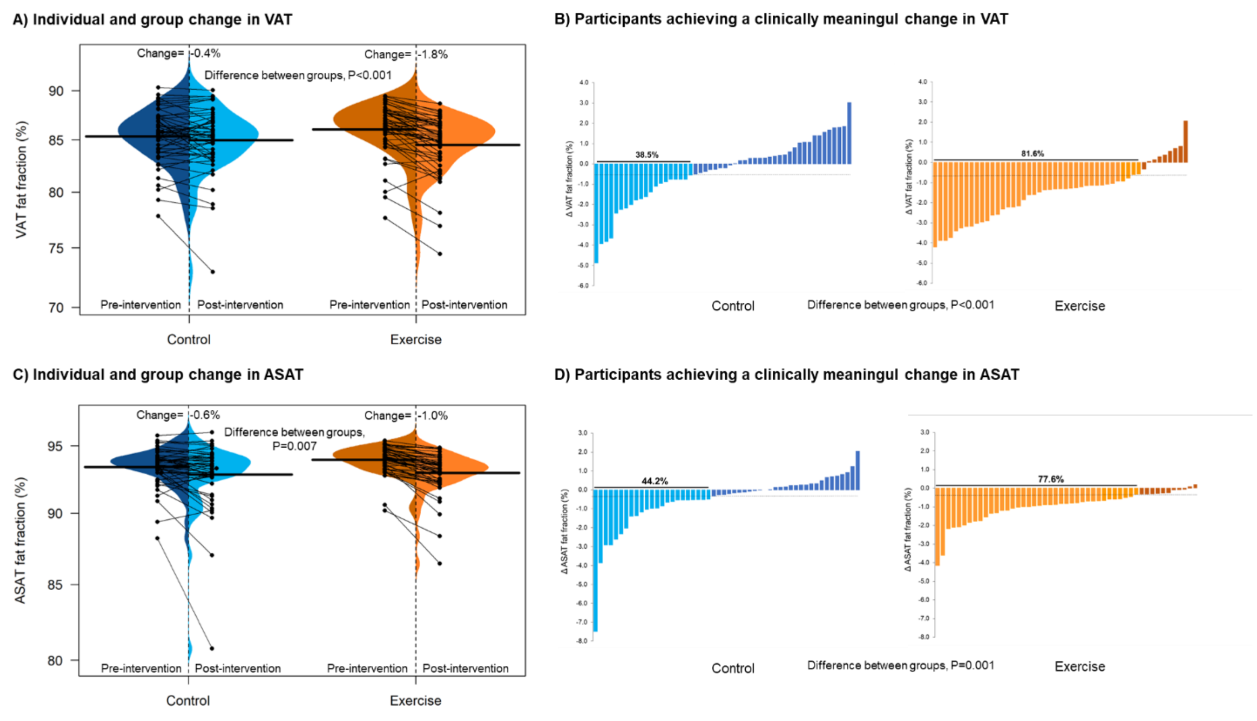

Changes were calculated as post- *minus* pre-intervention values. Analyses were adjusted for baseline values, age, and sex. Data analyses were conducted under the *per protocol* principle, i.e., subjects had to attend at least 50% of the family-based and psychoeducation program sessions (in the exercise group, no minimum attendance of exercise sessions was required). The brighter blue and orange bars represent those subjects who experienced a clinically meaningful change (i.e., responders) from baseline to post-intervention (Cohen's d ≥ 0.2). The darker blue and orange bars represent those participants who did not experience a clinically meaningful change (Cohen's d <0.2). Differences between the control and exercise groups were examined using the Chi-squared test.

© 2022 Cadenas-Sanchez C et al. *JAMA Network Open*.

eFigure 2. Mediation Model to Determine Whether Changes in VAT Area Mediated Changes in Insulin Resistance (HOMA)

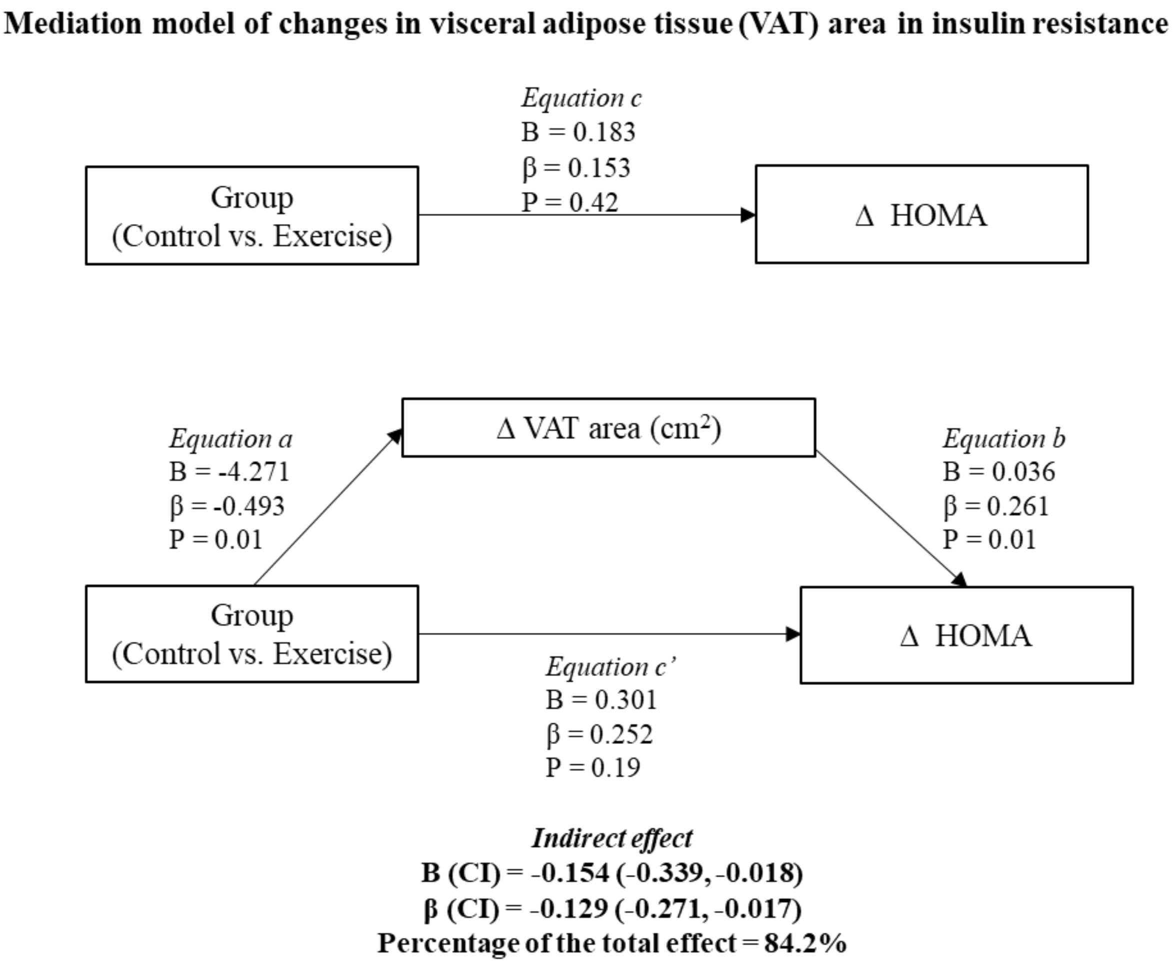

Sensitivity analysis using *per protocol* principle additionally including a minimum of 50% of attendance of the exercise sessions.

HOMA: homeostasis model assessment; VAT: Visceral adipose tissue.
Data presented are for *per protocol* analysis additionally including a minimum of 50% of attendance of the exercise group (n total = 97 participants; n =45 in the exercise group). Analyses were adjusted for baseline values, age, sex, and changes in height. Delta (Δ) expresses the outcome at post intervention with respect to baseline.

© 2022 Cadenas-Sanchez C et al. *JAMA Network Open*.

eFigure 3. Changes in Abdominal Visceral Adipose Tissue in Participants in the Lifestyle and Psychoeducation Program (Control Group) and in the Same Program Plus Supervised Exercise Training (Exercise Group) in Intention-to-Treat Analyses

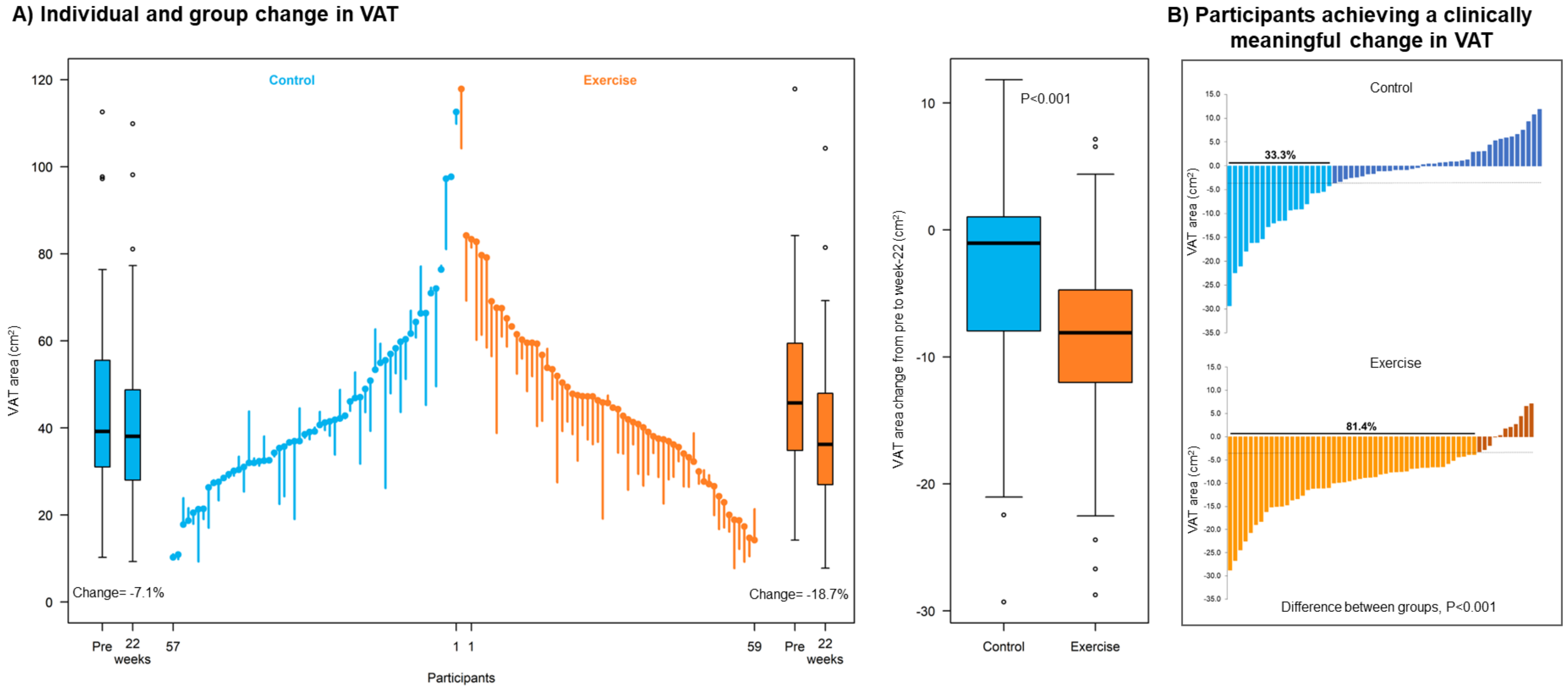

The ends of the boxes in the boxplots are located at the first and third quartiles, with the black line in the middle illustrating the median. Whiskers extend to the upper and lower adjacent values, the location of the furthest point within a distance of 1.5 interquartile ranges from the first and third quartiles. The parallel line plot (panel A) contains 1 vertical line for each participant which extends from their baseline to their 22-weeks value. Descending lines indicate a reduction in VAT. Pre-test values are placed in ascending order for the control group and descending order for the exercise group. Changes were calculated as post- *minus* pre-intervention values. Analyses were adjusted for baseline values, age, sex, and changes in height. Data analyses were conducted

© 2022 Cadenas-Sanchez C et al. *JAMA Network Open*.

under *intention-to-treat* analyses. In the last box (panel B), brighter blue and orange bars represent those participants who experienced a meaningful change (i.e., responders) from baseline to post-intervention ≥ 0.2 Cohen's *d*. Darker blue and orange bars represent those participants who did not experience a meaningful change ≥ 0.2 Cohen's *d* from baseline to post-intervention. Chi-square analyses were performed for examining differences between-group (i.e., control vs. exercise).

© 2022 Cadenas-Sanchez C et al. *JAMA Network Open*.

eFigure 4. Changes in Abdominal Subcutaneous Adipose Tissue, Intermuscular Abdominal, and Pancreatic Fat Fraction in Participants in the Lifestyle and Psychoeducation Program (Control Group) and in the Same Program Plus Supervised Exercise Training (Exercise Group) in Intention-to-Treat Analyses

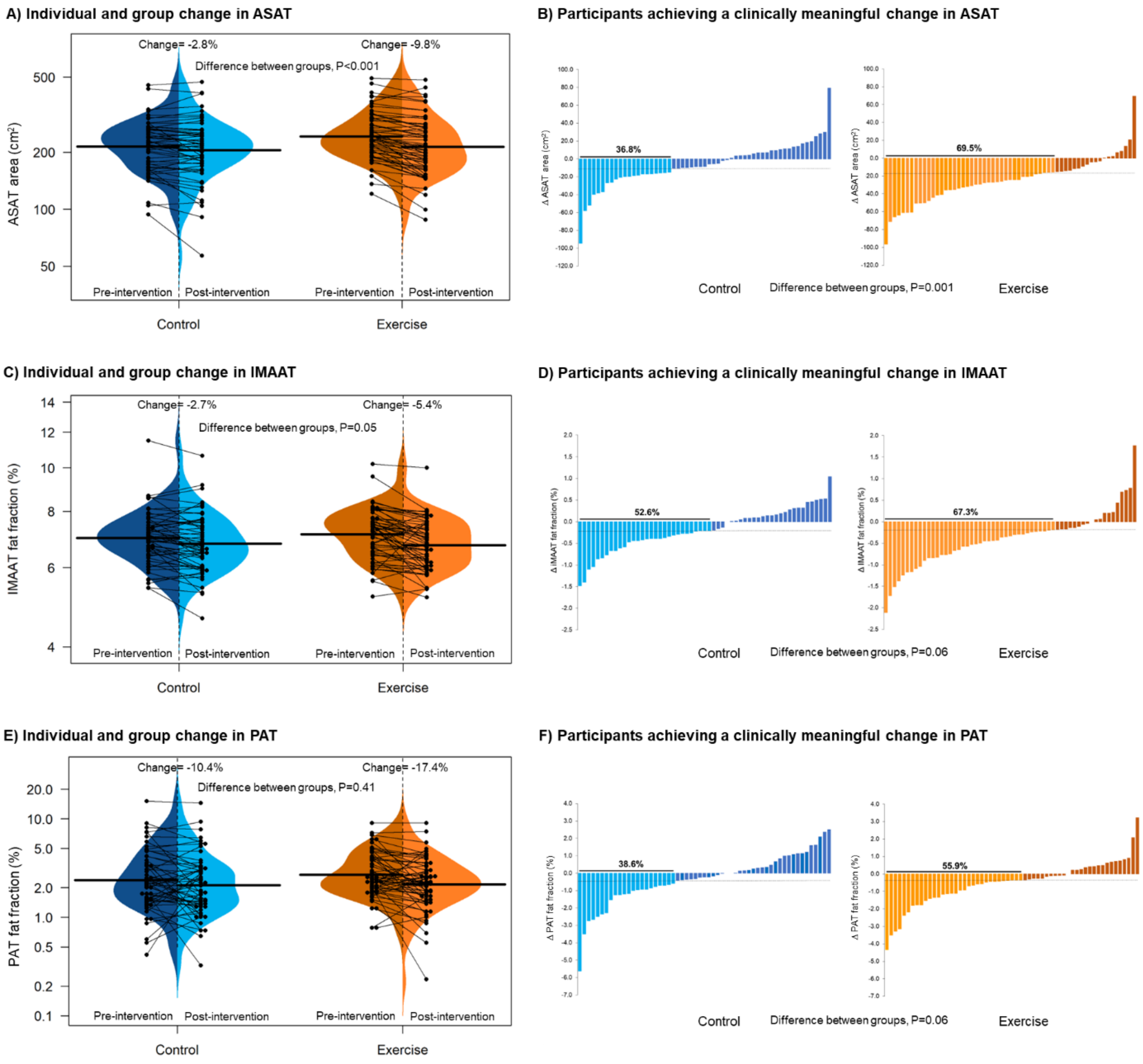

Changes were calculated as post- minus pre-intervention values. Analyses were adjusted for baseline values, age, and sex. For difference in ASAT area, an additional adjustment was made for changes in height. Data analyses were conducted under the *intention-to-treat* principle. The brighter blue and orange bars represent those subjects who experienced a clinically meaningful change (i.e., responders) from baseline to post-intervention (Cohen's d ≥ 0.2). The darker blue and orange bars represent those participants who did not experience a clinically meaningful change (Cohen's d <0.2). Differences between the control and exercise groups were examined using the Chi-squared test.

© 2022 Cadenas-Sanchez C et al. *JAMA Network Open*.

eFigure 5. Mediation Model to Determine Whether Changes in VAT Area Mediated Changes in Insulin Resistance (HOMA) in Intention-to-Treat Analyses

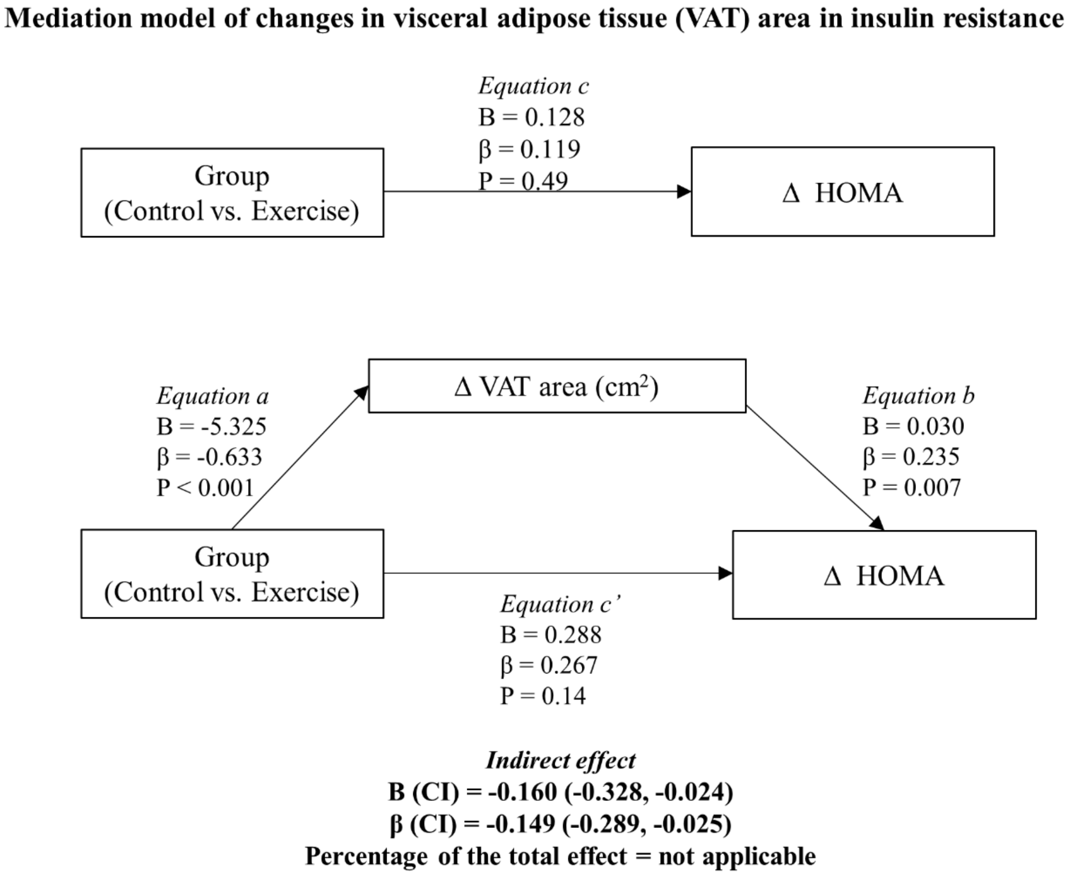

HOMA: homeostasis model assessment; VAT: Visceral adipose tissue.
Data presented are for *intention-to-treat* analysis. Analyses were adjusted for baseline values, age, sex, and changes in height. Delta (Δ) expresses the outcome at post intervention with respect to baseline.

© 2022 Cadenas-Sanchez C et al. *JAMA Network Open*.

eFigure 6. Mediation Model to Determine Whether Changes in VAT Area Mediated Changes in Insulin Resistance (HOMA) in Sensitivity Analysis Using Only Those Children Who Were Randomized

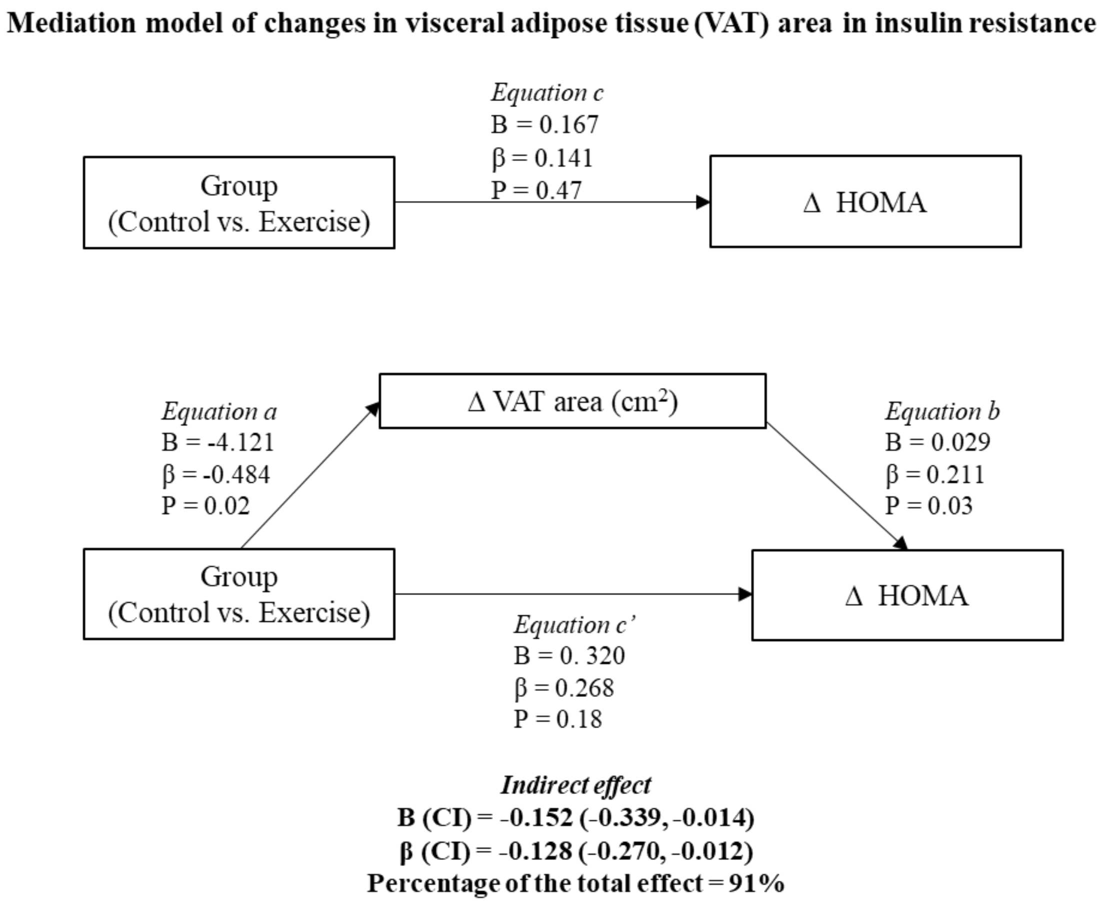

HOMA: homeostasis model assessment; VAT: Visceral adipose tissue.
Data are shown excluding those children/parents who were not randomized (n=11, all of them from the control group); therefore, only those randomized participants were included in the analyses.
Data presented are for *per protocol* analysis. Analyses were adjusted for baseline values, age, sex, and changes in height. Delta (Δ) expresses the outcome at post intervention with respect to baseline.

© 2022 Cadenas-Sanchez C et al. *JAMA Network Open*.
