# Supplementary material for: Effects of a Family-Based Lifestyle Intervention Plus Supervised Exercise Training on Abdominal Fat Depots in Children With Overweight or Obesity: A Secondary Analysis of a Nonrandomized Clinical Trial
Source: JAMA Netw Open. 2022 Nov 28;5(11):e2243864. doi: 10.1001/jamanetworkopen.2022.43864 (PMC9706365; doi:10.1001/jamanetworkopen.2022.43864)
Supplement: Supplement 3. — Data Sharing Statement [file jamanetwopen-e2243864-s003.pdf]

Data Sharing Statement

Cadenas-Sanchez. Effects of a Family-Based Lifestyle Intervention Plus Supervised Exercise Training on Abdominal Fat Depots in Children With Overweight or Obesity. *JAMA Netw Open*. Published November 28, 2022. doi:10.1001/jamanetworkopen.2022.43864

Data
Data available: No
Explanation for why data are not available: We did not obtain children's parents consent to widely share the data, nor was it included in the IRB protocol.
